# Dietary Changes of Youth during the COVID-19 Pandemic: A Systematic Review^☆^

**DOI:** 10.1016/j.tjnut.2024.02.022

**Published:** 2024-02-24

**Authors:** Nicolas Woods, Jamie A Seabrook, Holly Schaafsma, Shauna Burke, Trish Tucker, Jason Gilliland

**Affiliations:** 1 School of Health Studies, the University of Western Ontario, London, Ontario, Canada; 2 Human Environments Analysis Laboratory, the University of Western Ontario, London, Ontario, Canada; 3 Department of Epidemiology & Biostatistics, the University of Western Ontario, London, Ontario, Canada; 4 School of Food and Nutritional Sciences, Brescia University College, London, Ontario, Canada; 5 Children’s Health Research Institute, London, Ontario, Canada; 6 Lawson Health Research Institute, London, Ontario, Canada; 7 Department of Paediatrics, the University of Western Ontario, London, Ontario, Canada; 8 School of Occupational Therapy, the University of Western Ontario, London, Ontario, Canada; 9 Department of Geography and Environment, the University of Western Ontario, London, Ontario, Canada; 10 Health and Rehabilitation Sciences, Faculty of Health Sciences, the University of Western Ontario, London, Ontario, Canada

**Keywords:** nutrition, COVID-19, teen, children, youth

## Abstract

**Background:**

The coronavirus disease 2019 (COVID-19) pandemic has taken the lives of millions and disrupted countless more worldwide. Simply living through the pandemic has had drastic effects on the health of citizens. Diet, an important aspect of health, has been uniquely affected by the pandemic, although these changes have not been sufficiently studied among youth.

**Objectives:**

The objective of this systematic review was to investigate dietary changes of youth during COVID-19.

**Methods:**

A prespecified literature review was conducted using MEDLINE, EMBASE, Scopus, and CINAHL to identify studies from January 2020 to May 2023 that assessed dietary changes among youth aged ≤20 y compared with before the pandemic. Only quantitative observational studies that were published in English were included. Two authors completed all screening/study selection independently, with disagreements being resolved via discussion. Data extraction was completed by 1 author. Dietary changes were categorized into food groups and habits for analysis purposes.

**Results:**

In total, 67 studies met inclusion criteria. Most studies used recall to assess changes (48/67; 71.6%). Most studies found an increase in fruits and vegetables (24/46; 52.2%), grain products (6/11; 54.5%), meat, poultry, and eggs (4/8, 50.0%), diet quality indices and/or overall dietary assessments (7/13, 53.8%), and the frequency of snacking (9/12; 75.0%), whereas generally finding a decrease in ultraprocessed foods (32/53; 60.4%), compared with before the COVID-19 pandemic. Mixed findings or primarily no changes were found for fish and aquatic products, legumes, beans, seeds and nuts, milk and milk products, breakfast consumption, and nutrient intake.

**Conclusions:**

Mostly favorable dietary changes appear to have occurred among youth during COVID-19, although there were several mixed findings and unclear takeaways among the foods and habits under study. The heterogeneity of defining food groups was a noted limitation in the current review.

## Introduction

The COVID-19 is responsible for ∼6.9 million deaths since the WHO first declared it a pandemic on March 11, 2020 [1]. Because of the severity and infectious nature of this disease, more than one-third of countries worldwide began implementing restrictive measures for citizens in March 2020, in hopes to slow the spread [2]. In the months following, many countries began adopting policies, such as mandatory masking indoors, capacity limits of buildings, shifting work and school to online formats, and temporary closure of nonessential businesses, such as restaurants, bars, and recreational facilities [[3], [4], [5], [6]]. Although these restrictions have been effective at reducing the spread of the COVID-19 virus, there have been unintended effects of the pandemic on both the mental and physical health of individuals of all ages.

In a systematic review on the impact of the COVID-19 pandemic on the mental health of adolescents worldwide, living through the pandemic was found to be associated with higher anxiety, depression, and psychological distress, compared with prior [7]. A separate systematic review found that the pandemic has also been associated with reduced physical activity in youth, compared with prior [8]. Beyond these effects, the COVID-19 pandemic has brought on other negative changes for families as well, including loss of employment [9], reduced food security [10,11], and increased substance use, particularly alcohol [12].

Although these changes are important to investigate, dietary changes during the COVID-19 pandemic are especially important to consider, given the association that dietary intake has with risk of future chronic disease [13]. Potential reasons for dietary changes during the COVID-19 pandemic are numerous and include interruptions to the global food supply chain [14], increased boredom because of temporary stay-at-home measures [15], reduced food security [11], and increased sedentariness/screen time [16]. It is likely that these changes have collectively influenced a shift in dietary changes of people worldwide affected by the pandemic, coupled with the stress and anxiety that come with living through unprecedented times.

Most research investigating the impact of the COVID-19 pandemic on dietary intake has focused mostly on an adult population, with 2 previous reviews finding mostly unfavorable changes, including an increased consumption of alcohol, snacking, and ultraprocessed foods compared with before the COVID-19 pandemic [17,18]. It is important to note however, that youth are also susceptible to the effects of the pandemic [19]. Childhood and adolescence are critical periods of growth, as adults who developed food skills during their childhood and teen years have been shown to have more confidence regarding their cooking and food skills, cooking practices, attitudes, and diet quality, compared with those who did not develop these skills at these ages [20,21]. If the COVID-19 pandemic has negatively affected dietary habits among youth, these unhealthy habits and reliance on foods that require little cooking/preparation skills (e.g., ultraprocessed foods) may continue postpandemic and carry forward into adulthood, putting them at risk of chronic diseases, such as cardiovascular disease, obesity, various cancers, type 2 diabetes, and depression [[22], [23], [24]].

In a recent systematic review, authors investigated eating habits of children and adolescents during lockdowns globally, finding mixed results, such as increases in consumption of home-cooked meals, fruits, vegetables, and legumes, but also increases in foods, such as french fries and sweets [25]. Although this paper contributes to the knowledge of how youth’s diets have changed during COVID-19, it is limited in that it only considers dietary changes during “lockdowns,” which are heterogeneous in nature and vary in definition, by region. The COVID-19 pandemic has had drastic effects on a global scale, and although lockdowns have certainly contributed to these changes, the impacts which the COVID-19 pandemic has had extend beyond these lockdowns. Given the risk of chronic disease that has been routinely associated with poor diet quality [26], and the critical time-period which youth have to develop their food skills [20,21], it is vital that this population be investigated to see how the entirety of the COVID-19 pandemic has impacted their diets. As such, the purpose of this systematic review was to examine the dietary changes of youth (≤20 y) during the COVID-19 pandemic, compared with before the pandemic.

## Methods

The protocol for this systematic review is registered on PROSPERO (#412766) and was conducted in accordance with the PRISMA statement [27].

### Information sources

Searches were conducted by one author (NW) using MEDLINE (Ovid), EMBASE (Ovid), CINAHL, and Scopus. The search was conducted in May 2023, using a combination of keywords and section headers, after consultation with an academic librarian who helped refine the search. Papers were limited to those published from January 2020 to May 2023. The search strategy for each database can be found in Supplemental Table 1.

### Inclusion/exclusion criteria

Quantitative observational studies were included if they investigated dietary changes during the COVID-19 pandemic (compared with before the pandemic) among youth aged ≤20 y and were published in English. The timing of both “before the pandemic” and “during the pandemic” was left up to authors’ discretions; however, all dates of data collection ranged from March 2018 to April 2022. Dietary changes included shifts in food and/or nutrient intake, frequency of meals/snacks, diet quality scores, frequency of takeout food consumption, and intake of certain foods/food groups (e.g., fruits and vegetables, milk products). For younger ages, parents’ perceptions of their child’s food changes were recorded, although studies, which investigated total household changes and gave no description about youth changes were excluded. Studies were also excluded if they investigated dietary intake during the COVID-19 pandemic but did not inquire about dietary intake before the COVID-19 pandemic. Studies were included if the sample included age ranges beyond the one under study (i.e., >20 y), permitting that data for youth were stratified for, or in cases where data were not stratified, if the mean/median age was ≤20 y. Studies were excluded if the sample was limited to youth with a specific disease (e.g., inflammatory bowel disease), or those involving inpatient settings and/or other health services recruitment. Intervention studies were also excluded for the purposes of this review, as only dietary changes because of COVID-19 were investigated. Qualitative studies were also excluded if no quantitative component was included.

### Study selection

**FIGURE 1:**
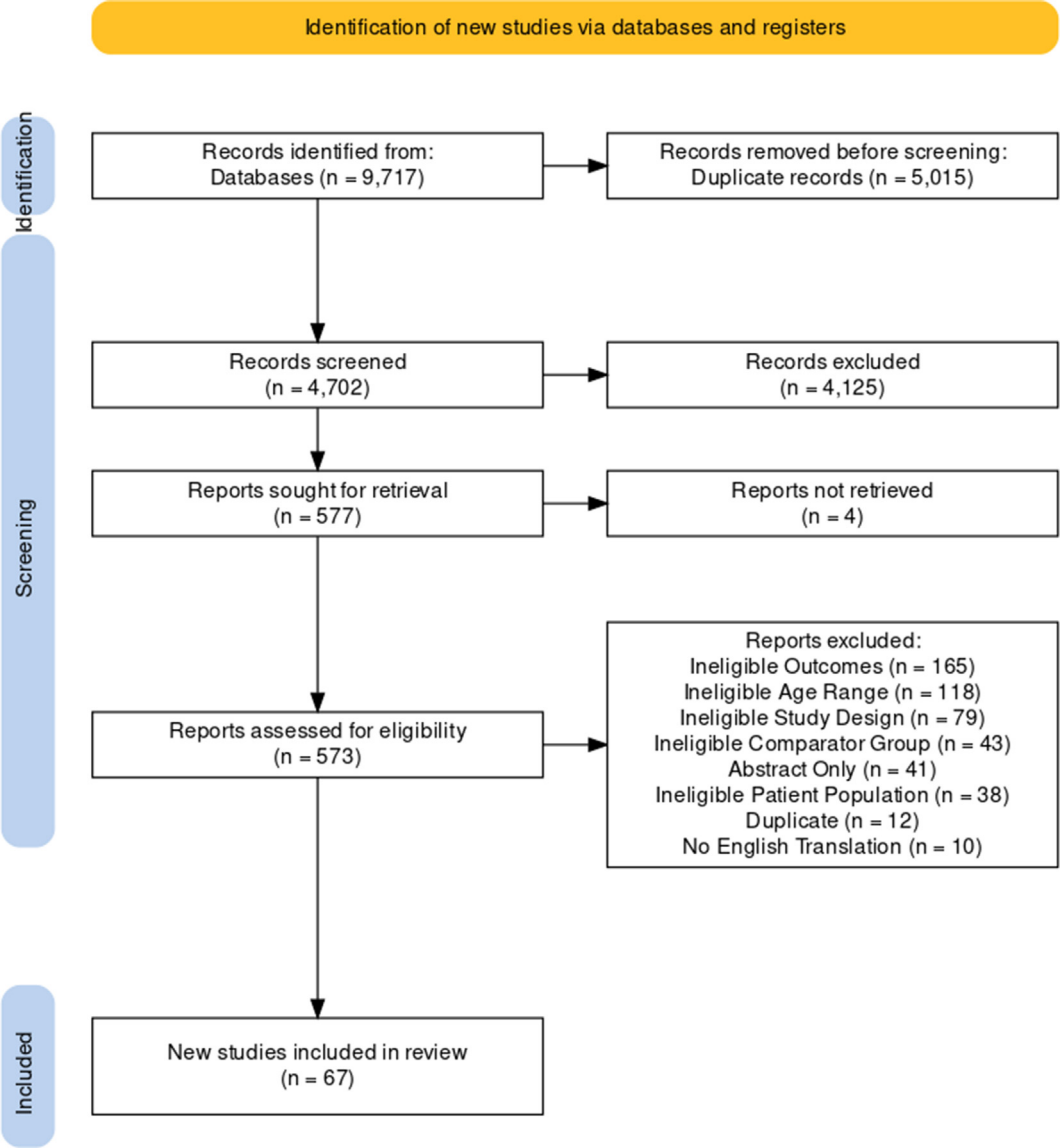
PRISMA flow diagram of included studies.

Results of the searches were exported via a research information systems file and imported into COVIDENCE Systematic Review Software for screening [28]. Two reviewers (NW and HS) completed the title and abstract screening based on the inclusion and exclusion criteria. Reviewers first completed this step independently and then resolved disagreements via discussion using COVIDENCE [28], which flagged conflicting responses. Duplicates were excluded before screening. Next, full-text screening was completed independently by both reviewers, for all articles that were selected for inclusion from the previous stage. Disagreement was again resolved using discussion. Articles were selected for inclusion, or excluded with reasons, which can be found within the PRISMA diagram (Figure 1).

### Data extraction

Data extraction was completed by 1 author (NW) using a custom created extraction chart that included: authors, number of participants, sample size, age range (or mean age ± SD, when the range was not given), gender/sex distribution, the method used to measure dietary changes, the dietary measures investigated (e.g., food groups, diet quality scores), the country the data were derived from, dates of data collection, method of reporting (parent compared with self-reported), method of comparison (prepandemic measurement compared with participant recall), and key findings of the articles. The Human Development Index (HDI), designed by the United Nations to represent a nation’s level of development on a scale of 0–1, was determined based on the countries studied, and a value was applied to each country based on the 2021 report (the most recent at time of writing) [29]. Any information not provided within the articles was treated as missing.

### Food group measures

Individual foods were categorized into food groups to analyze changes from a macro-level standpoint and to clarify and synthesize results. The following food groups were used: “Fruits and Vegetables,” “Fish and Aquatic Products,” “Grain Products,” “Legumes, Beans, Seeds, and Nuts,” “Meat, Poultry, and Eggs,” “Milk and Milk Products,” and “Ultra-Processed Foods.” These food groups were created by 1 registered dietitian (NW) to summarize findings and were based on pre-existing, commonly used food groups, such as those used in “Eating Well with Canada’s Food Guide” [30] and the “Mediterranean Diet Quality Index Questionnaire” [31]. Combinations of 2 or more food groups, or measures of food intake changes that did not apply to any of the above food groups, were not classified. “Diet Quality Indices and Overall Assessments,” “Nutrients,” “Breakfast,” and “Snacking” were also assessed (despite not fitting in the above food groups), because of the number of studies investigating these habits. The grouping of these categories can be found in Supplemental Table 2.

### Data synthesizing

Each food or dietary habit within a study was assigned to its overarching group (e.g. Fruits and Vegetables and Snacking). If the study found an increase in at least one of these foods within that category, the study was classified as finding an increase, and the same applies if a decrease was found. If none of the foods within a group changed, the study was treated as finding no difference. If the study found both an increase and decrease of foods/habits from a specific group, the study was treated as finding both an increase and decrease for that group. Finally, studies which found changes (either an increase or decrease), but did not present statistical significance, were still treated as changes, whereas those that did present statistical significance were treated as changes only if significance was met. However, each change (or lack thereof) was noted as either a statistically significant one or not, to avoid biasing findings based on descriptive studies.

### Quality assessment

Studies were graded on their quality using the National Heart, Lung, and Blood Institute’s Quality Assessment Tool for Observational Cohort, and Cross-Sectional Studies (National Heart, Lung, and Blood Institute, 2021), based on 14 different scoring metrics. Inherent to the current review’s objectives, 4 of these scoring metrics were not relevant as they pertained to varying levels of the exposure, measurement of exposure, and the blinding of the exposure measurement. As the current study’s exposure variable is living through the COVID-19 pandemic, these were not included, and only 10 scoring metrics were used to assess quality. Studies were given a total score out of 10 (1 for each category), dependent on how many criteria each met. Two authors (NW and HS) completed the quality assessment independently for all studies, with disagreements resolved via discussion. To ensure consistency with overall findings, each domain of dietary changes was assessed, stratified by study quality (see Supplemental Table 3). For the purposes of this subanalysis, studies with a score of 6 or higher were deemed “high quality,” scores of 4–5 were deemed “medium quality,” and those with a score of 3 or lower were deemed “low quality.” Dietary changes among each level of study quality were compared on the number of studies suggesting an increase, decrease, or no change.

## Results

A total of 4702 articles were originally retrieved for screening. On completion of title, abstract, and full-text screening, 67 studies were deemed eligible for inclusion (Figure 1). The median number of youth participants across studies was 782.5 (range: 50–109, 282), and ages of youth ranged from 1 to 20 y. Dates under study ranged from March 2018 (prepandemic reference) to April 2022 (during pandemic), although most studies collected data during January–December 2020. More information on characteristics of the studies can be found in Table 1 [[32], [33], [34], [35], [36], [37], [38], [39], [40], [41], [42], [43], [44], [45], [46], [47], [48], [49], [50], [51], [52], [53], [54], [55], [56], [57], [58], [59], [60], [61], [62], [63], [64], [65], [66], [67], [68], [69], [70], [71], [72], [73], [74], [75], [76], [77], [78], [79], [80], [81], [82], [83], [84], [85], [86], [87], [88], [89], [90], [91], [92], [93], [94], [95], [96], [97], [98]], and a summary of the quality assessment can be found in Table 2.

**TABLE 1:** Summary of studies

Author	Number of youth	Gender/sex distribution	Age (range, or mean ± SD)	Method of measuring dietary changes	Dietary changes investigated	Country, (HDI)	Dates of data collection	Main findings	Method of reporting	Method of comparison for prior dietary intake
Aguilar-Martínez et al. (2021) [32]	303	70% Girls	14–18 y	FFQ/questionnaire	Cereals, legumes, fruit, vegetables, dairy products, meat, fish, eggs, convenience food, sweets and pastries, soft drinks, amount of food, variety of foods, snacks between meals, fresh food, and packaged food	Spain, (0.90)	October 2019–July 2020	More youth increased their consumption of fresh food, snacks between meals, variety of foods, amount of food, eggs, dairy products, vegetables, fruit, and cereals, compared with before confinement, than decreased. More youth decreased their consumption of packaged food, soft drinks, sweets and pastries, convenience food, fish, meat, and legumes than increased	Self-reported	Prepandemic measurement (longitudinal)
Al Hourani et al. (2021) [33]	477	52% Female	6–17 y	FFQ/questionnaire	Milk, labaneh, creamy cheese, cooked vegetables, raw vegetables, fruits, fresh fruits, 100% fruit juice, bread, rice, macaroni and pasta, cornflakes, red meat, chicken, fish, boiled eggs, carbonated beverages, canned juice, tea, coffee, French fries, pastries (fatayer), pizza, potato chips, popcorn, sugar, ice cream, cake, chocolate bar, and Arabic sweets	Jordan (0.72)	June 2020	Milk and milk products, vegetables, fruits, bread, rice, red meat, chicken, boiled eggs, carbonated beverages, canned juice, tea, coffee, French fries, pastries, pizza, potato chips, popcorn, sugar, ice cream, cake, chocolate bar and Arabic sweets increased in one or both age groups, compared with before lockdown	Children 6–12: Parent-reported; Teens 13–17: Self-reported	Dietary recall
Alfayez et al. (2022) [34]	432	61.6% Females	6–18 y	FFQ/questionnaire	Number of meals, number of fruits and vegetables, red meat, fast food, chips packets, and soda drinks and energy drinks consumed	Saudi Arabia (0.88)	No info	Compared with prepandemic, there was a significant increase in number of meals, fruits and vegetables, red meat, fast food, chips packets, and soda and energy drinks	Self-reported	Dietary recall
Almutairi et al. (2022) [35]	1500	54.0% Females	11–15 y	FFQ/questionnaire	Fast food meals, unhealthy snacks, soft/energy/sport/fruit drinks, healthy food like fruit, vegetables, meat and milk, increase in food consumption	Saudi Arabia (0.88)	No info	Compared with pre-COVID-19 restrictions, more individuals increased than decreased their intake of healthy food like fruit, vegetables, meat and milk, and food consumption. More individuals decreased their intake of fast food meals, unhealthy snacks, and soft/energy/sport/fruit drinks.	Self-reported	Dietary recall
Androutsos et al. (2021) [36]	397	49% Girls	2–18 y	FFQ/questionnaire	Salty snacks, fruits and fresh juices, vegetables, prepacked juices and sodas, dairy, red meat, poultry, fish, pasta, legumes, sweets, total snacks, fast food, and breakfast	Greece (0.89)	April–May 2020	Fruits and fresh juices, vegetables, dairy, pasta, sweets, total snacks, and breakfast significantly increased during lockdown, compared with prior. Fast food significantly decreased compared with prior.	Parent-reported	Dietary recall
Angoff et al. (2022) [37]	140	No info	No info	FFQ/questionnaire	Child eats dessert every day, child gets frequent snacks, snacks after dinner, packaged snacks (e.g., granola bars, chips), breakfast	United States (0.92)	July 2020–August 2020	Compared with prepandemic, children eating desserts every day, children getting frequent snacks, snacks after dinner, and packaged snacks decreased more than increased. Breakfast increased more than decreased.	Parent-reported	Dietary recall
Baghlaf et al. (2022) [38]	417	No info	6–11 y	FFQ/questionnaire	Soft drinks, fruit juice, flavored milk	Saudi Arabia (0.88)		Compared with prepandemic, soft drinks, fruit juice, and flavored milk decreased more than increased.	Parent-reported	Dietary recall
Bahatheg (2021) [39]	330	52% Female	4–7 y	FFQ/questionnaire	Vegetables, fruits, chocolate or sweets, unhealthy food, small meals, cakes, biscuits and cupcakes, frozen foods (e.g., pizza, nuggets, pies), sweetened juices, and milk	Saudi Arabia (0.88), United Kingdom (0.93), Turkey (0.84)	No info	Majority of participants not eating more vegetables, fruits, chocolate or sweets, cakes, biscuits, and cupcakes, frozen foods (e.g., pizza, nuggets, pies), sweetened juices, and milk during lockdown, compared with prior. Majority of participants eating more small meals, compared with prior	Parent-reported	Dietary recall
Bekelman et al. (2022) [40]	347	47% Female	4–12 y	FFQ/questionnaire	Quantity of food, SSBs, Discretionary food	United States (0.92)	July 2019–March 2021	No significant changes	Self-reported and Parent-reported	Prepandemic measurement (longitudinal)
Weihrauch-Blüher et al. (2023) [41]	1004	52.3% Girls	3–17 y	FFQ/questionnaire	Eating more healthy	Germany (0.94)	March 2022–April 2022	Compared with before the pandemic, parents reported more children eating less healthy than more healthy.	Parent-reported	Dietary recall
Borger et al. (2021) [42]	434	No info	5–6 y	24-h dietary recall	Healthy Eating Index-2015 (HEI-2015) score and subcomponents, calories, fiber, calcium, vitamin D, potassium, added sugars, sodium, and saturated fat	United States (0.92)	April 2019–June 2020	Total vegetables, and saturated fat significantly decreased in the post-emergency declaration (ED) group, compared with the pre-ED group. Sodium, refined grains, vitamin D, and saturated fat (% kcals) significantly increased in the post-ED group, compared with the pre-ED group. Among intra-individual differences, total vegetables significantly decreased more during post-ED, compared with pre-ED. Sodium and calories significantly increased more during post-ED, than pre-ED.	Parent-reported	Prepandemic measurement (longitudinal and non-longitudinal)
Burkart et al. (2022) [43]	74	47% Female	7–12 y	FFQ/questionnaire	Healthy foods, and unhealthy foods	United States (0.92)	Spring 2019–Summer 2020	Healthy and unhealthy foods significantly increased in Spring 2020 compared with Spring 2019. Healthy foods significantly increased from Summer 2020, compared with Summer 2019.	Parent-reported	Prepandemic measurement (longitudinal)
Calabriano et al. (2022) [44]	416	62.1% Girls	10–19 y	FFQ/questionnaire	Breakfast, lunch, legumes, vegetables, fruits, fries, candies, sugary drinks, ultraprocessed, fast food	Chile (0.86)	May-July 2020	Compared with pre-confinement, breakfast, and lunch significantly increased. Legumes, vegetables, fruits, fries, candies, sugary drinks, ultraprocessed foods, and fast food significantly decreased.	Self-reported	Dietary recall
Carroll et al. (2020) [45]	310	No info	5.7 ± 2.0 y	FFQ/questionnaire	Quantity of food, fruits and vegetables, snack foods, and fast food/take out	Canada (0.94)	April–May 2020	51% of children’s eating habits changed. Among these children, more reported an increase in quantity of food, fruits and vegetables, and snack foods compared with before the pandemic, than reported a decrease. More children reported a decrease in fast food/takeout than an increase.	Parent-reported	Dietary recall
Cui et al. (2021) [46]	3542	52% Female	11.4 ± 1.6 y	24-h Dietary recall	Grains, tubers, vegetables, fruit, meats, eggs, aquatic products, soybean products and nuts, milk and milk products, and overall dietary diversity score (DDS)	China (0.77)	November 2019–November 2020	Overall DDS scores, aquatic products, soybean products and nuts were significantly lower in 2020, compared with prepandemic. Milk and milk products were significantly higher compared with prepandemic.	Self-reported	Prepandemic measurement (longitudinal)
Díaz-Rodríguez et al. (2022) [47]	478	Unknown	0–6 y	FFQ/questionnaire	Eating habits, snacks, fast food and high-calorie foods, impulsive eating,	Spain (0.90)	April–May 2020	Compared with before lockdown, eating habits improved more than worsened, and snacks, fast food and high-calorie foods increased more than decreased.	Parent-reported	Dietary recall
Dondi et al. (2021) [48]	5811	No info	0–18 y	FFQ/questionnaire	Food intake, snacks (junk food), fruit juices, and soft drinks	Italy (0.88)	September–October 2020	More parents reported their children eating more than eating less, compared with before the pandemic. Among those eating more, 60.3% reported more snacks, 14.0% reported more fruit juices, and 10.4% reported more soft drinks	Parent-reported	Dietary recall
Dragun et al. (2021) [49]	324	72% Female	14–18 y	FFQ/questionnaire	Fruits, vegetables, meat and processed meat, and sweets and snacks	Croatia (0.86)	April 2018–May 2020	More youth increased the frequency of fruits, vegetables, meat and processed meat than decreased, compared with before lockdown. More youth decreased their intake of sweets and snacks than increased.	Self-reported	Dietary recall
Gardner et al. (2022) [50]	983	54.8% Girls	11–17 y	FFQ/questionnaire	Sugar-sweetened beverage consumption, discretionary food intake, fruit, vegetable	Australia (0.95)	July 2019–October 2021	Compared with prepandemic, sugar-sweetened beverage, discretionary food intake, fruit, and vegetable intake decreased.	Self-reported	Prepandemic measurement (longitudinal)
Gedeon et al. (2022) [51]	4001	No info	5–11 y	FFQ/questionnaire	Skipping one of the main meals (breakfast, lunch, dinner), snacking between meals, quantity/portions of meals and snacks, fruits and vegetables, balanced diet (including health ingredients such as whole wheat, pulses, legumes, eggs, nuts, fruits and vegetables), junk food/fast food and fried food, sugar-sweetened beverages (carbonated soft drinks, sugar-sweetened juices), sweets/ candies/ chocolate, unhealthy food when he/she is bored or stressed or upset, immunity-boosting foods (lemon, turmeric, garlic, citrus fruits and green leafy vegetables), nutrition supplements to boost immunity, support in eating healthy	Lebanon (0.71)	March–April 2022	Compared with prepandemic, more children decreased than increased their intake of fruits and vegetables, balanced diet, junk food/fast food and fried food, sugar-sweetened beverages, and their intake of nutrition supplements to boost immunity. More children increased than decreased their frequency of not skipping one of the main meals, intake of snacking between meals, sweets/candies/chocolate, unhealthy food when he/she is bored or stressed or upset, immunity-boosting foods, and support in eating healthy.	Parent-reported	Dietary recall
Hanbazaza et al. (2021) [52]	280	49% Girls	6–15 y	FFQ/questionnaire	Vegetables, fruits, dairy products, soft drinks, sugar-sweetened beverages, sweets/candy/chips, fast food, number of meals, and breakfast consumption	Saudi Arabia (0.88)	June–July 2020	Number of meals, dairy products, fast food and breakfast decreased during COVID-19 curfew, compared with prior.	Parent-reported	Dietary recall
Hashem et al. (2020) [53]	765	47% Female	4–16 y	FFQ/questionnaire	Sweets and unhealthy food, regular protein intake, snacks between meals, and late snacks during night	Egypt (0.73)	May 2020	More youth did not increase, or it was pre-existing before lockdown, their intake of sweets and unhealthy food, regular protein intake, and snacks between meals. More youth had late snacks during night after lockdown, compared with prior.	Parent-reported	Dietary recall
He et al. (2022) [54]	5963	50.1% Girls	10.7 ± 2.2 y	FFQ/questionnaire	Sugar-sweetened beverages	China (0.77)	December 2019–July 2020	Compared with prepandemic, consumption of sugar-sweetened beverages significantly decreased during the pandemic.	Self-reported	Prepandemic measurement (longitudinal)
Horikawa et al. (2021) [55]	1111	51% Girls	10–14 y	FFQ/questionnaire	Milk and dairy products, meat, fish, or eggs, vegetables, and fruits	Japan (0.92)	December 2020	Milk and dairy products, meat, fish, or eggs, vegetables, and fruits all decreased during the state of emergency, compared with prior.	Parent-reported	Dietary recall
Husain et al. (2022) [56]	1148	47.65% Female	7 mo–3 y	FFQ/questionnaire	Dietary score, cereals and potatoes, nuts and pulses, fish and meat, eggs, milk and dairy products, fruits that are yellow or orange inside, green leafy vegetables, other fruits and vegetables	India (0.63)	January–November 2020	Dietary score, cereals and potatoes, nuts and pulses, and yellow/orange fruits significantly increased from pre-lockdown to lockdown. Fish and meat, milk and dairy products significantly decreased.	Parent-reported	Prepandemic measurement (longitudinal)
James et al. (2021) [57]	2218	48% Girls	8–11 y	FFQ/questionnaire	Breakfast, fizzy drink, sugary snack, takeaway, and fruit/vegetable	United Kingdom (0.93)	March 2019–June 2020	Breakfast and sugary snack consumption increased during school closures compared with 2019. Takeaway food consumption decreased, compared with prior.	Self-reported	Prepandemic measurement (non-longitudinal)
Jia et al. (2021) [58]	2,824	76% Female	17.5 ± 1.2 y	FFQ/questionnaire	Rice, wheat products, other staple foods, meat, poultry, fish, eggs, sugar-sweetened beverages, coffee and caffeinated drinks, tea, other beverages, fresh vegetables, preserved vegetables, fresh fruit, soybean products, and dairy products	China (0.77)	May 2020	Wheat products, and tea increased, compared with before lockdown. Rice, meat, poultry, fish, eggs, fresh fruit, soybean products, dairy products, and sugar-sweetened beverages decreased.	Self-reported	Dietary recall
Kalyoncu et al. (2021) [59]	328	52% Girls	8–18 y	FFQ/questionnaire	Fresh fruits, fresh vegetables, fast food, carbonated beverages, and packaged food consumption	Turkey (0.84)	May 2020	More youth reported an increase in fresh fruits consumption, and fresh vegetables consumption than a decrease during the COVID-19 outbreak, compared with prior. More youth reported a decrease in fast food consumption, carbonated beverages, and packaged food consumption than an increase compared with prior.	Parent-reported	Dietary recall
Kang et al. (2022) [60]	109, 282	48.1% Girls	12–18 y	FFQ/questionnaire	Fruit and fast food consumption	Korea (0.92)	June 2019–November 2020	Fruit consumption significantly decreased during the pandemic, compared with prior.	Self-reported	Prepandemic measurement (non-longitudinal)
Kim (2022) [61]	54, 848	48.3% Female	15.1 ± 1.7 y	FFQ/questionnaire	Breakfast	Korea (0.92)	April 2021	Compared with before COVID-19, breakfast skipping increased more than it decreased.	Self-reported	Dietary recall
Kim et al. (2021) [62]	105,600	48% Female	12–18 y	FFQ/questionnaire	Breakfast, fruit, soda drink, sweet drinks, and fast food	Korea (0.92)	June 2019–November 2020	Fruit, fast food, soda drink, sweet drinks and breakfast were decreased significantly during 2020, compared with 2019.	Self-reported	Prepandemic measurement (non-longitudinal)
Kołota and Głąbska (2021) [63]	1334	53% Girls	10–16 y	FFQ/questionnaire	Fruits, vegetables, soft drinks, French fried potatoes, and fast food	Poland (0.88)	June 2020	Fruits, and vegetables significantly increased during remote education, compared with prior.	Self-reported	Dietary recall
Kołota and Głąbska, (2021) [64]	1334	53% Girls	10–16 y	FFQ/questionnaire	Takeaway meals, fried foods, fat, sugar, vegetables/salad, dessert/pudding, fruit, sausages/burgers, fruit/vegetables, sweet snacks, and healthy diet	Poland (0.88)	June 2020	Trying to keep overall fat intake down, trying to keep overall sugar intake down, having one serving of vegetables (excluding potatoes) or salad with an evening meal, having at least one serving of fruits per day, having one serving of vegetables or salad a day, having plenty of fruit and vegetables, choosing fruit as a snack between meals, and having at least 3 servings of fruit most days increased during COVID-19, compared with prior.	Self-reported	Dietary recall
Konstantinou et al. (2021) [65]	1509	48% Female	5–14 y	FFQ/questionnaire	Fish, food items that contain sugar, salty/savory foods (snacks), breakfast, ready-made food, supplements, fruits, vegetables, legumes, and meat	Cyprus (0.90)	June–July 2020	Food items that contained sugar, and breakfast increased in post-lockdown, compared with before lockdown**.** Salty/savory foods (snacks), supplements, and ready-made food decreased in post-lockdown. Fish significantly changed, but it's unclear how.	Parent-reported	Dietary recall
Kyan et al. (2022) [66]	1491	51.4% Females	12–18 y	FFQ/questionnaire	Breakfast	Japan (0.92)	2019–2021	Breakfast consumption did not significantly differ between 2019 and 2021.	Self-reported	Prepandemic measurement (non-longitudinal)
Lee et al. (2022) [67]	46, 475	48.0% Female	12–18 y	FFQ/questionnaire	Breakfast	Korea (0.92)	June 2019–November 2020	Compared with prepandemic, breakfast consumption did not significantly differ among both females and males.	Self-reported	Prepandemic measurement (non-longitudinal)
Lee et al. (2022) [68]	800	47.1% Girls	12–18 y	24-h dietary recall	Calories, Fruit, Vegetables, calcium, vitamin A, sodium, fat, sugar, skipping breakfast, soft drinks, eating out	Korea (0.92)	2019–2020	Compared with prepandemic, there was a significant increase in soft drink consumption, and a significant decrease in eating out.	Self-reported	Prepandemic measurement (non-longitudinal)
López-Bueno et al. (2020) [69]	860	49% Girls	3–16 y	FFQ/questionnaire	Fruits and vegetables	Spain (0.90)	March–May 2020	Fruits and vegetables decreased among those 3–5 y, and those 6–12 y, compared with before confinement.	Parent-reported	Dietary recall
Luo et al. (2022) [70]	2,824	76% Female	17.5 ± 1.2 y	FFQ/questionnaire	Takeaway food	China (0.77)	May 2020	Weekly frequency of food ordering decreased during lockdown, when compared with before COVID-19 restrictions.	Self-reported	Dietary recall
Malta et al. (2021) [71]	9470	50% Female	12–17 y	FFQ/questionnaire	Fruit, vegetables, frozen foods, chocolates and sweets, and potato chips	Brazil (0.75)	June–October 2020	Regular consumption of vegetables, inadequate consumption of frozen foods, and chocolates and sweets increased during social distancing, compared with prior. Inadequate consumption of potato chips decreased, compared with prior.	Self-reported	Dietary recall
Mastorci et al. (2021) [72]	1289	52% Female	10–14 y	FFQ/questionnaire	Mediterranean Diet Quality Index for children and adolescents (KIDMED)	Italy (0.88)	September 2019–April 2020	Total KIDMED scored significantly increased during quarantine, compared with standard conditions.	Self-reported	Prepandemic measurement (longitudinal)
Maximova et al. (2022) [73]	1095	51% Girls	9–12 y	FFQ/questionnaire	Number of meals, and number of snacks	Canada (0.94)	November 2020–February 2021	More youth increased the number of meals and snacks than decreased it during school closures, compared with before school closures.	Self-reported	Dietary recall
McNicholas et al. (2022) [74]	1371	No info	0–12 y (6.8 ± 3.2)	FFQ/questionnaire	Quantity of food, healthy foods (fruits and vegetables), unhealthy foods (potato chips, chocolate biscuits and cake, and soft drink, cordial or juice)	Australia (0.95)	June 2020	Compared with prepandemic, parents agreed more than disagreed that their child increased their intake of quantity of food, healthy foods (fruits and vegetables), and unhealthy foods (potato chips, biscuits and cake, and soft drink, cordial or juice).	Parent-reported	Dietary recall
Medrano et al. (2021) [75]	113	49% Girls	8–16 y	FFQ/questionnaire	Mediterranean Diet Quality Index for children and adolescents (KIDMED)	Spain (0.90)	September 2019–April 2020	Total KIDMED score significantly increased during confinement, compared with prior.	Self-reported	Prepandemic measurement (longitudinal)
Mikulec et al. (2022) [76]	330	69.1% Girls	7–18 y	FFQ/questionnaire	Number of meals, coffee, tea, sweet snacks, salty snacks	Poland (0.88)	September–October 2022	Compared with prepandemic, coffee, tea, sweet snacks, and salty snacks increased.	Self-reported	Dietary recall
Moitra et al. (2022) [77]	1298	46.7% Girls	13.2 ± 1.2	FFQ/questionnaire	Carbonated beverages, fast food, fried snack, high salt food, high sugar food, green leafy vegetable, fruits, breakfast	India (0.63)	January–March 2021	Compared with before the pandemic, carbonated beverages, fast food, fried snack, high salt food, high sugar food, green leafy vegetable, fruit increased more than decreased. Breakfast decreased more than increased.	Self-reported	Dietary recall
Munasinghe et al. (2020) [78]	582	80% Female	13–19 y	24-h dietary recall	Fruits, vegetables, and fast food	Australia (0.95)	November 2019–April 2020	Fast food consumption was significantly decreased from pre-implementation to postimplementation of physical distancing guidelines.	Self-reported	Prepandemic measurement (longitudinal)
Nanayakkara et al. (2022) [79]	118	No info	6–18 y	FFQ/questionnaire	Quantity of food, perceived healthiness	Australia (0.95)	August–November 2020	Compared with before the pandemic, more parents reported an increase in quantity of food, and perceived healthiness, than a decrease.	Parent-reported	Dietary recall
Ng et al. (2021) [80]	3440	54% Girls	11–15 y	FFQ/questionnaire	Fruits, vegetables, sweets, soft drinks, and energy drinks	Czech Republic (0.89)	June 2020	More youth increased their intake of fruits and vegetables more than decreased, during lockdown compared with prior. Among the most frequent consumers of sweets, soft drinks and energy drinks, more increased their intake of these foods than decreased during lockdown, compared with prior. Among the least frequent consumers of these foods, more decreased their intake than increased, compared with prior.	Self-reported	Dietary recall
Perrar et al. (2022) [81]	108	42% Female	3–18 y	3-d weighed diet records	Calories, fat, protein, carbohydrate, free sugar, ultraprocessed foods, fruits and vegetables, sugar-sweetened beverages, and juices	Germany (0.94)	March 2018–Aug. 2020	Calories decreased significantly during the pandemic, compared with prepandemic intake.	Parents or older participants	Prepandemic measurement (longitudinal)
Philippe et al. (2021) [82]	498	52% Girls	3–12 y	FFQ/questionnaire	Candy, chocolate, fruit juice, soda, chips, salty biscuits, ice cream, pastries, cake, sweet cookies, cream desserts, milks, yogurt, cheese, quark, fresh and dried fruits, nuts, bread, sandwich, pizza, savory pies, cheese, cereals, cereal bars, and compote, fruits in syrup	France (0.90)	April–May 2020	Candy, chocolate, fruit juice, soda, chips, salty biscuits, ice cream, pastries, cake, sweet cookies, cream dessert, milks, yogurt, cheese, quark, fresh and dried fruits, and nuts increased during lockdown, compared with prior. Compote, fruits in syrup decreased.	Parent-reported	Dietary recall
Pujia et al. (2021) [83]	439	44% Girls	5–14 y	FFQ/questionnaire	Milk, cheese and yogurt, meat, fish and eggs, processed meat, pasta and rice, bread, pizza and bakery products, vegetables, legumes and fruits, oil, butter and margarine, sweet beverages, ice cream and desserts, sweet packaged snacks, candy, and chocolate	Italy (0.88)	September 2020–April 2021	Milk, cheese and yogurt, meat, fish and eggs, processed meat, pasta and rice, bread, pizza and bakery products, vegetables, legumes and fruits, ice cream and desserts, sweet packaged snacks, and chocolate had more individuals increase their intake than decrease during lockdown, compared with prior. Oil, butter and margarine, sweet beverages, and candy had more individuals reduce their intake than increase during lockdown.	Parent-reported	Dietary recall
Radwan et al. (2021) [84]	2398	78% Girls	6–18 y	FFQ/questionnaire	Restaurant, takeaway and delivery, fast food meals or snacks, fruit, vegetables, soda or glasses of sweet tea, beans, chicken, or fish, regular snack chips or crackers, desserts and other sweets, and margarine, butter, or meat fat to season vegetables or put on potatoes, bread, or corn	Palestine (0.72)	August 2020–September 2020	Vegetables, and beans, chicken, or fish increased during the pandemic, compared with prior. Restaurant, takeaway, and delivery, fast food meals or snacks, fruit, soda or glasses of sweet tea, regular snack chips or crackers, desserts and other sweets, and margarine, butter, or meat fat to season vegetables or put on potatoes, bread, or corn decreased, compared with prior.	Under 12: Parent-reported; Over 12: Self-reported	Dietary recall
Ramos-Álvarez et al. (2021) [85]	50	34% Girls	11–12 y	FFQ/questionnaire	Vegetables dishes or salad, fruit, bread, high saturated fat food, dairy, fish, soft drinks, pulses, sweets and jelly beans, savory snacks, and cakes and pastries	Spain (0.91)	October 2019–May 2020	Fruits, and savory snacks increased during lockdown, compared with prior. Dairy decreased, compared with prior	Self-reported	Prepandemic measurement (longitudinal)
Robinson et al. (2023) [86]	2011	49.0% Female	5–17 y	FFQ/questionnaire	Unhealthy food, fruit and vegetables	Australia (0.95)	June–September 2020	Compared with pre-lockdown, unhealthy food, and fruits and vegetables increased more than decreased.	Parent-reported	Dietary recall
Rucińska et al. (2022) [87]	486	49.8% Female	3–18 y	FFQ/questionnaire	Sweets, Fast food, number of meals (per day)	Poland (0.88)	April 2020	Number of meals, and sweets significantly increased. Fast food significantly decreased.	Parent-reported	Dietary recall
Ruiz-Roso et al. (2020) [88]	820	61% Girls	10–19 y	FFQ/questionnaire	Legumes, vegetables, fruits, sweet food, fried food, processed meat, sugar-sweetened beverages, and fast food	Spain (0.90) Italy (0.90) Brazil (0.75) Colombia (0.75) Chile (0.86)	April–May 2020	Legumes, vegetables, fruit intake, sweet food, and fried food significantly increased during confinement, compared with prior. Fast food significantly decreased.	Self-reported	Dietary recall
Saltaouras et al. (2022) [89]	397	No info	2–18 y	FFQ/questionnaire	Breakfast, lunch, dinner, fast food	Greece (0.89)	March–May 2020	Compared with before lockdown, breakfast significantly increased. Fast food significantly decreased	Parent-reported	Dietary recall
Schwarzová et al. (2023) [90]	252	50.0% Girls	6–14 y	FFQ/questionnaire	Meals, snacks, white bread and pastry, bread and pastry-wholemeal, cereal products, Muesli and breakfast cereals - sweet, potatoes, vegetable, vegetable fresh, fruit, fruit fresh, milk and dairy products, cheese, meat and meat products, poultry, fish, legumes, eggs, sweets, fast food, nuts, honey	Slovakia (0.85)	March 2020–June 2021	Compared with before the pandemic, meals, snacks, white bread and pastry, potatoes, vegetable fresh, fruit, fruit fresh, cheese, fish, eggs, sweets, honey, and snacks significantly increased during the pandemic.	Self-reported	Dietary recall
Segre et al. (2021) [91]	82	46% Female	6–14 y	FFQ/questionnaire	Eating as normal, junk food and sweets, and quantity and quality of food	Italy (0.90)	May–June 2020	More teens not eating as normal, eating more junk food and sweets during quarantine, compared with prior. More teens were not eating different quantity and quality of food compared with prior	Self-reported	Dietary recall
Shenoy et al. (2021) [92]	463	50% Female	4–16 y	FFQ/questionnaire	Number of meals, vegetables, fruits, junk food, and sugary drinks	India (0.63)	No info	Number of meals per day, vegetable intake, and fruit intake increased during lockdown, compared with before. Junk food intake decreased compared with before lockdown	Parent-reported	Dietary recall
Skolmowska et al. (2021) [93]	2448	No info	15–20 y	FFQ/questionnaire	Takeaway meals, fried foods, fat, sugar, vegetables/salad, dessert/pudding, fruit, sausages/burgers, fruit/vegetables, sweet snacks, and healthy diet	Poland (0.88)	April–May 2020	Takeaway meals, fried foods, sugar, and dessert/puddings decreased during the pandemic, compared with prior. Vegetables/salads, and fruits increased compared with prior	Self-reported	Dietary recall
Szczepańska et al. (2022) [94]	1098	No info	4–8 y	FFQ/questionnaire	Number of meals, vegetables, fruits, sweets (cakes, cookies, candies), salty snacks (chips, peanuts, sticks), Fast food products (kebab, pizza, hamburger), and sweetened drinks (coca cola, flavored waters)	Poland (0.88)	May 2020	Number of meals, fruits, sweets, salty snacks, and sweetened drinks significantly increased during lockdown, compared with prior. Fast food products significantly decreased.	Parent-reported	Dietary recall
Thiab et al. (2022) [95]	230	45.2% girls	6–16 y	FFQ/questionnaire	Healthy meals (balanced meals including fruit, vegetables, protein...etc.), unhealthy meals (fast food, sweets, fizzy drinks, chips...etc.)	Jordan (0.72)	August 2020–January 2021	Healthy and unhealthy meals significantly increased during quarantine, compared with prior.	Parent-reported	Dietary recall
Van den Broek et al. (2023) [96]	306	52.5% Female	14.3 ± 0.6	FFQ/questionnaire	Sugar-sweetened beverages, sweet snacks, savory snacks, fruits and vegetables	Netherlands (0.94)	Spring 2019–Fall 2020	Compared with before lockdown, SSBs, savory snacks, and fruits and vegetables significantly decreased.	Self-Reported	Prepandemic measurement (longitudinal)
Villodres et al. (2021) [97]	899	54% Girls	10–14 y	FFQ/questionnaire	Mediterranean Diet Quality Index for children and adolescents (KIDMED)	Spain (0.90)	February–April 2021	Mediterranean diet adherence was not significantly different during COVID-19, compared with prior	Self-reported	Dietary recall
Wang et al. (2021) [98]	1795	52% Girls	10–19 y	FFQ/questionnaire	Staples, pulses, fruits, vegetables, and animal-source foods	Ethiopia (0.50), Burkina Faso (0.45), Nigeria (0.54)	July–November 2020	Staples, pulses, fruits, vegetables, and animal-source foods decreased more than increased during the pandemic, compared with prior.	Self-reported	Dietary recall

Abbreviation: FFQ, food frequency questionnaire.

**TABLE 2:** Quality assessment (highest to lowest ranked studies)

Author and year	Objective stated?	Study population defined?	Participation of eligible persons >50%?	Were subjects recruited from the same population?	Sample size justification?	Exposure measured before outcomes?	Timeframe long enough?	Were the outcome measures defined, valid, and reliable?	Loss to follow-up 20% or less?	Were key potential confounding variables adjusted for?	Quality rating (max score of 10)
Perrar et al., 2022 [81]	✓	✓	✓	✓	X	✓	✓	✓	✓	✓	9
Burkart et al., 2022 [43]	✓	✓	✓	✓	X	✓	✓	✓	X	✓	8
Medrano et al., 2021 [75]	✓	✓	X	✓	✓	✓	✓	✓	X	✓	8
Ramos-Álvarez et al., 2021 [85]	✓	✓	✓	✓	X	✓	✓	✓	✓	X	8
He et al., 2022 [54]	✓	✓	✓	✓	X	✓	✓	X	✓	X	7
Lee et al., 2022 [68]	✓	✓	✓	✓	X	✓	✓	✓	N/A	X	7
Bekelman et al., 2022 [40]	✓	✓	X	✓	X	✓	✓	✓	X	X	6
Borger et al., 2021 [42]	✓	✓	N/A	✓	X	✓	✓	✓	N/A	X	6
Cui et al., 2021 [46]	X	✓	X	X	X	✓	✓	✓	✓	✓	6
Gardner et al., 2022 [50]	✓	✓	X	✓	X	✓	✓	✓	X	X	6
Husain et al., 2022 [56]	X	✓	X	✓	X	✓	✓	✓	✓	X	6
Kim et al., 2021 [62]	✓	✓	X	X	X	✓	✓	✓	N/A	✓	6
Kołota and Głąbska, 2021 [63]	✓	✓	X	✓	✓	X	X	✓	N/A	✓	6
Mastorci et al., 2021) [72]	✓	✓	X	✓	X	✓	✓	✓	X	X	6
Munasinghe et al., 2020 [78]	✓	✓	X	✓	X	✓	✓	✓	X	X	6
Van den Broek et al., 2023 [96]	✓	✓	X	✓	X	✓	✓	✓	N/A	X	6
Aguilar-Martínez et al., 2021 [32]	✓	✓	X	✓	X	X	X	✓	N/A	✓	5
Dragun et al., 2021 [49]	✓	✓	✓	✓	X	X	X	✓	N/A	X	5
Gedeon et al., 2022 [51]	✓	✓	X	✓	✓	X	X	✓	N/A	X	5
James et al., 2021 [57]	✓	✓	X	X	X	✓	✓	✓	N/A	X	5
Kang et al., 2022 [60]	✓	✓	X	✓	X	✓	✓	X	N/A	X	5
Kołota and Głąbska, 2021 [64]	✓	✓	X	✓	X	X	X	✓	N/A	✓	5
Lee et al., 2022 [68]	✓	✓	✓	✓	X	X	X	✓	N/A	X	5
Luo et al., 2022 [70]	✓	✓	X	✓	X	✓	✓	X	N/A	X	5
Maximova et al., 2022 [73]	✓	✓	✓	✓	X	X	X	X	N/A	✓	5
Moitra et al., 2022 [77]	✓	✓	X	✓	✓	X	X	✓	N/A	X	5
Baghlaf et al., 2022 [38]	✓	X	X	✓	✓	X	X	✓	N/A	X	4
Weihrauch-Blüher et al., 2023 [41]	✓	✓	✓	✓	X	X	X	X	N/A	X	4
Carroll et al., 2020 [45]	✓	✓	✓	✓	X	X	X	X	N/A	X	4
Hanbazaza and Wazzan, 2021 [52]	✓	✓	X	✓	✓	X	X	X	N/A	X	4
Jia et al., 2021 [58]	✓	✓	X	✓	X	X	X	✓	N/A	X	4
Kalyoncu et al., 2021 [59]	✓	X	✓	✓	X	X	X	✓	N/A	X	4
Kim, 2022 [61]	✓	✓	✓	✓	X	X	X	X	N/A	X	4
Kyan et al., 2023 [66]	✓	✓	X	X	X	✓	✓	X	N/A	X	4
Philippe et al., 2021 [82]	✓	X	X	✓	X	X	X	✓	N/A	✓	4
Robinson et al., 2023 [86]	✓	✓	✓	✓	X	X	X	X	N/A	X	4
Ruiz-Roso et al., 2020 [88]	✓	✓	X	✓	X	X	X	✓	N/A	X	4
Skolmowska et al., 2021 [93]	✓	✓	X	✓	X	X	X	✓	N/A	X	4
Villodres et al., 2021 [97]	✓	X	X	✓	X	X	X	✓	N/A	✓	4
Al Hourani et al., 2022 [33]	✓	X	X	✓	X	X	X	✓	N/A	X	3
Alfayez et al., 2022 [34]	✓	X	X	✓	✓	X	X	X	N/A	X	3
Almutairi et al., 2022 [35]	✓	✓	✓	X	X	X	X	X	N/A	X	3
Horikawa et al., 2021 [55]	✓	✓	X	✓	X	X	X	X	N/A	X	3
Konstantinou et al., 2021 [65]	✓	✓	X	✓	X	X	X	X	N/A	X	3
López-Bueno et al., 2020 [69]	✓	X	X	✓	X	X	X	X	N/A	✓	3
McNicholas et al., 2022 [74]	✓	✓	X	✓	X	X	X	X	N/A	X	3
Ng et al., 2021 [80]	✓	✓	X	✓	X	X	X	X	N/A	X	3
Pujia et al., 2021 [83]	✓	✓	X	X	✓	X	X	X	N/A	X	3
Rucińska et al. (2022) [87]	✓	✓	X	X	✓	X	X	X	N/A	X	3
Segre et al., 2021 [91]	✓	✓	X	✓	X	X	X	X	N/A	X	3
Bahatheg 2021 [39]	✓	X	X	X	X	X	X	✓	N/A	X	2
Calabriano et al., 2022 [44]	✓	X	X	X	X	X	X	✓	N/A	X	2
Díaz-Rodríguez et al., 2022 [47]	✓	X	X	X	✓	X	X	X	N/A	X	2
Radwan et al., 2021 [84]	✓	X	X	✓	X	X	X	X	N/A	X	2
Saltaouras et al., 2022 [89]	✓	✓	X	X	X	X	X	X	N/A	X	2
Szczepańska et al., 2022 [94]	✓	X	X	✓	X	X	X	X	X	X	2
Thiab et al., 2022 [95]	✓	X	X	X	✓	X	X	X	N/A	X	2
Wang et al., 2021 [98]	✓	✓	X	X	X	X	X	X	N/A	X	2
Androutsos et al., 2021 [36]	✓	X	X	X	X	X	X	X	N/A	X	1
Angoff et al., 2022 [37]	✓	X	X	X	X	X	X	X	N/A	X	1
Dondi et al., 2021 [48]	✓	X	X	X	X	X	X	X	N/A	X	1
Hashem et al., 2020 [53]	✓	X	X	X	X	X	X	X	N/A	X	1
Malta et al., 2021 [71]	✓	X	X	X	X	X	X	X	N/A	X	1
Mikulec et al., 2022 [76]	✓	X	X	X	X	X	X	X	N/A	X	1
Nanayakkara et al., 2022 [79]	✓	X	X	X	X	X	X	X	N/A	X	1
Schwarzová et al., 2023 [90]	✓	X	X	X	X	X	X	X	N/A	X	1
Shenoy et al., 2021 [92]	X	X	X	X	✓	X	X	X	N/A	X	1

### Method of food intake assessment

Most studies used either a food frequency questionnaire or alternative questionnaire to inquire about dietary changes (62/67; 92.5%). A 3-d weighed diet record was used by 1 study, whereas a 24-h dietary recall was used by 4 studies. Prepandemic measurements of dietary intake were used as comparison tools in 20 studies, and among these studies, 14 used the same cohort of youth for both measurements, whereas 7 used a separate group of youth for the 2 timepoints (1 study used both). Most studies (*n* = 47) relied on participant recall comparing dietary intake before and during COVID-19.

### Country of study/HDI

A total of 31 different countries were included in this review. Spain (*n* = 7), Poland (*n* = 6), and the United States (*n* = 6) were the 3 most studied countries, whereas most studies came from within Europe (*n* = 31). Other countries included Australia, Korea, Saudi Arabia, Italy (*n* = 5 for each), China, India (*n* = 4 for both), Brazil, Germany, Greece, United Kingdom, Canada, Chile, Japan, Jordan, Turkey (*n* = 2 for each), Croatia, France, Czech Republic, Egypt, Cyprus, Palestine, Colombia, Ethiopia, Burkina Faso, Lebanon, Netherlands, Slovakia, and Nigeria (*n* = 1 for each). HDI values ranged from 0.45 (Burkina Faso) to 0.95 (Australia), although most countries where studies took place are classified by as “High” to “Very High” development (*n* = 63/67, 94.0%), based on their scores.

### Study quality

The median study quality score was 4 (out of 10), which was also the most common score. The study quality scores ranged from 1 to 9, although only 1 study received a 9/10 score. The most common reasons studies had lower scores were because they failed to disclose or did not have a participation rate of eligible people >50%, not providing a sample size justification, having a cross-sectional study design, and not controlling for important covariates in the analysis. Finally, subanalysis of each domain by study quality revealed largely that although results did not drastically change, higher quality studies were more likely to produce null findings (see Supplemental Table 3). Overall, study quality results are presented in Table 2.

## Changes in Food Consumption/Habits

**FIGURE 2:**
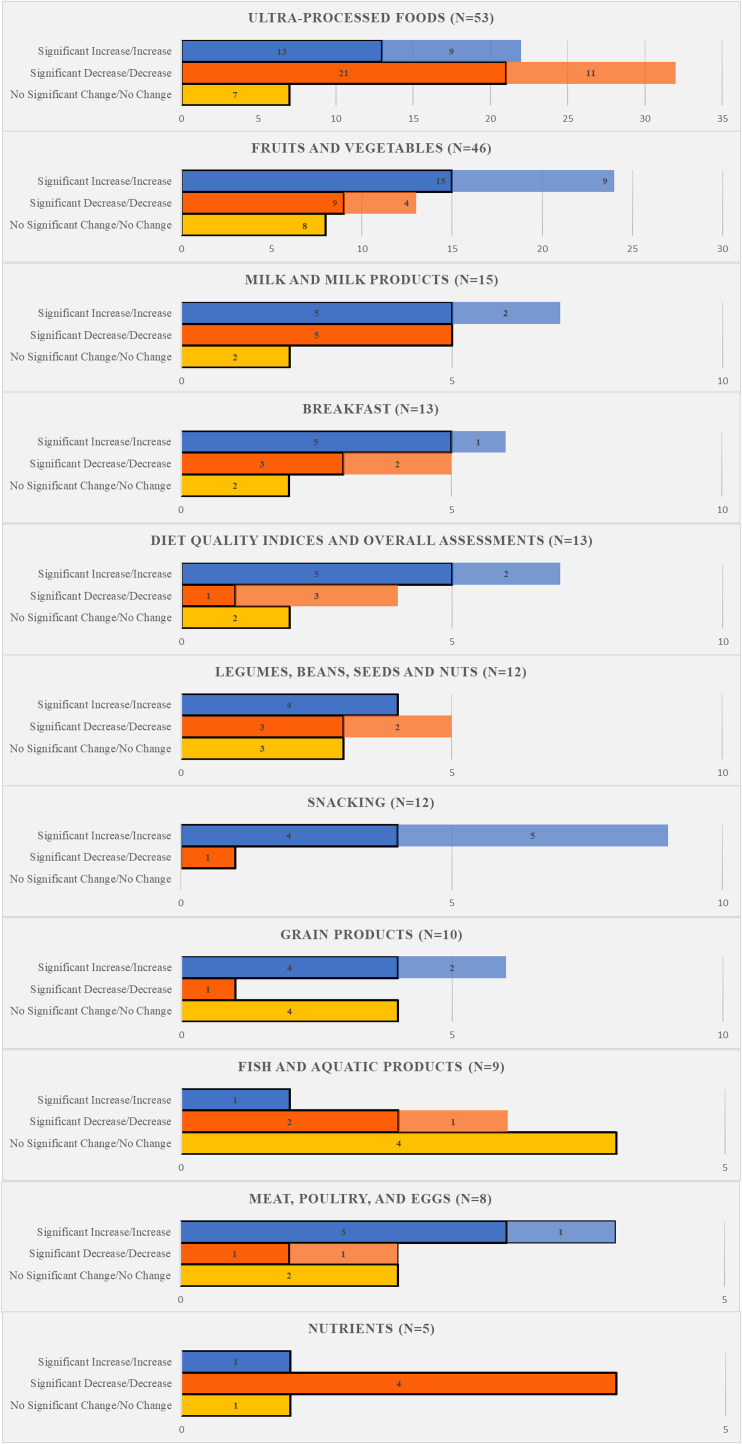
Summary of study findings. ∗Bolder colors indicate significant changes, whereas transparent ones represent changes of unknown statistical significance.

Overall study findings are summarized below (Figure 2).

### Ultraprocessed foods

Fifty-three (79.1%) studies investigated changes in ultraprocessed foods [[32], [33], [34], [35], [36], [37], [38], [39], [40],[43], [44], [45],[47], [48], [49], [50], [51], [52], [53], [54],[57], [58], [59], [60],[62], [63], [64], [65],^68^,^70^,^71^,[74], [75], [76], [77], [78],[80], [81], [82], [83], [84], [85], [86], [87], [88], [89], [90], [91], [92], [93], [94], [95], [96]]. The most studied foods within this category included “fast-food” (*n* = 15), “soft drinks” (*n* = 9), and “sugar-sweetened beverages” (*n* = 8). Among these 53 studies, 32 found a decreased consumption (11 nonsignificant [32,35,37,38,45,49,50,51,59,80,83], and 21 statistically significant [36,44,52,54,57,58,61,65,68,70,71,78,82,84,[87], [88], [89],[92], [93], [94],^96^]) in one or more foods within this category, whereas 22 found an increase (9 nonsignificant [47,51,74,76,77,80,83,86,91], and 13 statistically significant [33,34,36,43,57,68,71,82,87,88,90,94,95]), when compared with pre-COVID. Finally, there were 3 studies where intake of ultraprocessed foods changed, but it could not be determined whether the majority increased or decreased their intake [39,48,53].

### Fruits and vegetables

Forty-six (68.7%) studies investigated fruit or vegetable intake [[32], [33], [34],^36^,^38^,^39^,^42^,[44], [45], [46],[48], [49], [50], [51], [52],[55], [56], [57], [58], [59], [60],[62], [63], [64], [65],^68^,^69^,^71^,^74^,^75^,^77^,^78^,[80], [81], [82], [83], [84], [85], [86],^88^,^90^,[92], [93], [94],^96^,^98^]. The most common foods studied (within the “Fruits and Vegetables” group) were “fruits” (*n* = 30), “vegetables” (*n* = 24), and “fruits and vegetables” (*n* = 11). Among these 46 studies, 24 found an increase (9 nonsignificant [32,45,49,59,74,77,80,83,86] and 15 statistically significant [33,34,36,56,63,64,71,82,84,85,88,90,[92], [93], [94]]), compared with 13 that found a decrease (4 nonsignificant [38,50,51,98], and 9 statistically significant [36,47,77,80,82,86,[87], [88], [89]]), when compared with before COVID-19. One study found simultaneously, an increase and a decrease in one or more components of Fruits and Vegetables, whereas 2 studies found changes in consumption, but it could not be determined whether most participants increased or decreased their intake [39,48].

### Milk and milk products

Fifteen (22.4%) studies investigated changes in milk and/or milk products [32,33,36,39,42,46,52,55,56,58,75,82,83,85,90]. The most studied foods within this category included “dairy” (*n* = 5), and “milk” (*n* = 4). Seven studies found an increase in the consumption of milk and milk products (2 nonsignificant [32,83], and 5 statistically significant [33,36,46,82,90]), whereas 5 studies found a statistically significant decrease [52,55,56,58,85]. One study found a change in the consumption of milk and milk products, but it could not be determined whether most increased or decreased their intake [39].

### Legumes, beans, seeds, and nuts

Thirteen (19.4%) studies investigated changes in consumption of legumes, beans, seeds, and/or nuts [32,36,44,46,56,58,65,75,82,85,88,90,98]. “Legumes” made up the most commonly studied food of this group (*n* = 8), whereas “nuts” (*n* = 3) were the second most common. Four studies found a significant increase in legumes, beans, seeds, and/or nut consumption during COVID-19 compared with prior [56,75,82,88], whereas 5 found a decrease (2 nonsignificant [32,46] and 3 statistically significant [44,46,58]).

### Grain products

Eleven (16.4%) studies investigated changes in consumption of one or more grain products during COVID-19 [32,33,36,42,46,58,75,82,83,85,90], with the most common studied foods being “bread” (*n* = 3), pasta and rice,” and “rice” (*n* = 2 for both). Among these 11 studies, 6 found an increase (2 nonsignificant [32,83], and 4 statistically significant [33,36,42,58]) in at least 1 component of grain products during COVID-19 compared with prior, whereas 1 found a statistically significant decrease [58].

### Fish and aquatic products

Nine (13.4%) studies investigated “fish” (*n* = 8), or “aquatic products” (*n* = 1), which were the only foods within this category [32,33,36,46,58,65,75,85,90]. Among these 9 studies, 3 found a decrease (1 nonsignificant [32], and 2 statistically significant [46,58]), whereas 1 found a statistically significant increase [90]. Four of the 9 studies found no significant change. Finally, 1 study found a significant change in fish consumption, but it cannot be determined in what direction.

### Meat, poultry, and eggs

Eight (11.9%) studies investigated changes in meat, poultry, and/or eggs [32,33,34,36,56,58,65,90]. The most studied foods within this category included “eggs” (*n* = 5), “meat” (*n* = 4), and “poultry” and “red meat” (*n* = 3 for both). Among these 8 studies, 4 found an increase (1 nonsignificant [32], and 3 statistically significant [33,34,90]) in consumption during COVID-19, compared with prior, whereas 2 found a decrease (1 nonsignificant [32] and 1 statistically significant [58]).

### Other findings

Thirteen (19.4%) studies investigated diet quality indices and/or overall dietary assessments [41,42,[45], [46], [47],^51^,^56^,^72^,^75^,^85^,^93^,^97^]. Seven of these studies found an increase in diet quality (2 nonsignificant [47,79], and 5 statistically significant [56,72,75,85,93]), and 4 studies found a decrease (3 nonsignificant [41,45,51], and 1 statistically significant [46]). The most used diet quality index was Mediterranean Diet Adherence (KIDMED) score (*n* = 3), whereas “healthy diet” (*n* = 2) was the second most common method of assessing overall dietary changes.

Breakfast was investigated in 14 studies (20.9%), with 6 finding an increase (1 nonsignificant [37], and 5 statistically significant [36,44,57,75,89]), and 5 finding a decrease (2 nonsignificant [61,77], and 3 statistically significant [36,37,44,52,57,61,62,[65], [66], [67], [68],^75^,^77^,^89^]). Consumption of snacks was investigated in 12 studies (17.9%), with 9 finding an increase (5 nonsignificant [32,45,51,76,73], and 4 statistically significant [36,85,90,94]), and 2 finding a decrease (1 significant [96], and 1 nonsignificant [37]) compared with pre-COVID [32,36,37,45,51,53,73,76,85,90,94,96]. One study investigated changes in snacking, but it cannot be determined whether most increased or decreased [53].

Finally, nutrient intake changes were investigated in 5 studies (7.5%), and included calories, carbohydrate, sugar (including in the form of “added sugar,” and “free sugar”), fat, protein, fiber, calcium, vitamin A, vitamin D, potassium, sodium, fat, and saturated fat in 1 or more of the studies [42,64,68,81,93]. Most nutrients were not significantly different during the pandemic compared with prior, although findings included a significant decrease in sugar (*n* = 2) [64,93], total fat (*n* = 1) [64], and a significant increase in sodium (*n* = 1) [42] and vitamin D (*n* = 1) [42]. Mixed results were found for calories [42,81] and saturated fat [42], as studies suggested both an increase and decrease (*n* = 1 for each).

## Discussion

This review examined youth’s dietary changes during the COVID-19 pandemic, finding largely positive changes, beginning first with fruit and vegetable consumption. Most studies found an increase in fruits and vegetables, which is a positive change, as past research has shown that teens and younger children tend to not consume as many fruits and vegetables as recommended [[99], [100], [101]]. These results are somewhat conflicting with a similar review which found that most studies found a decrease in fruit and/or vegetable intake among individuals of all ages during COVID-19 restrictions, compared with prior [18]. One potential reason for this inconsistency is the differing populations investigated, as the prior review did not specifically study youth and included a mostly adult population. Second, even though this previous review found that most studies reported a decrease in fruit and vegetable consumption, the number of studies indicating a decrease was only slightly higher than the number suggesting an increase. (4–2, respectively) [18]. Finally, these findings are in agreement with another previous review on the cardiometabolic risk factors of youth during COVID-19 by Karatzi et al. [102], who found a general increase in fruit consumption compared with before COVID-19.

Possible explanations for the increase in fruit and vegetable consumption in the current review are numerous, with one being the increased time parents may have had to spend at home with their children positively influencing this. In a previous systematic review investigating change in home cooking/culinary practices, one noted side-effect of COVID-19 restrictions (among those making positive changes) was the increased time some parents were afforded to cook home meals [103]. In general, this may have caused an increase in fruit and vegetable consumption, because these foods often require time for preparation, which has been noted as a barrier to increasing produce consumption [104]. Another potential reason could be related to the method of recall, as a minority of studies in this review used a longitudinal design method. As a result, for the other studies, relying on recall is necessary to document changes, but is susceptible to recall bias [105] and social desirability bias [106]. Therefore, it is difficult to disentangle the impact which this may have had on the perceived increase of fruit and vegetable intake as youth (and their parents) may not be accurate in their recollections of intake.

Ultraprocessed foods were assessed by most studies in this review, and most found a decrease in consumption of 1 or more of these foods, compared with those which found an increase. Although again, increased parental oversight is a possible reason for the decrease, it should also be considered that during COVID-19 restrictions, some fast-food restaurants were severely restricted in how they were able to operate, which varied depending on governmental policies put in place [5]. As such, although this can be seen as a positive change, it may not necessarily have been a conscious decision on the part of youth and could partially be regarded as a direct consequence of COVID-19 restrictions. In this sense, it is unclear whether this decrease in consumption will continue as restrictions have largely been removed.

It should also be noted that these findings are in direct contrast with a previous systematic review on the topic which suggested that there was an overwhelming increase in unhealthy (“junk”) food during lockdowns among children and youth, compared with prior [25]. One reason for this discrepancy may be the differences in coding of food groups, as well as the current review containing additional studies that investigated ultraprocessed foods (when compared with both previous reviews). For example, Pourghazi et al. [25] incorporated “snacks” as inherently “junk foods,” which our review only did if the snack was clearly described as an ultraprocessed food. Another key difference is that our review classified almost 100 different foods as ultraprocessed foods (see Supplemental Table 1), whereas the previous review by Pourghazi et al. [25] listed 6 different food groups (snacks, fast foods, French fries or chips, processed food, and sweets) as “junk foods”, making direct comparisons between the categorization of the reviews more challenging, because it is unclear how these food groups are further broken down. As such, the differences in these groupings and slight differences in inclusion criteria may have contributed to the contrasting findings between the 2 reviews.

Next, although most studies which investigated intakes of legumes, beans, seeds, and nuts found that youth tended to decrease their intake during COVID-19, results were largely mixed, as a similar proportion found an increase, or no change at all. These foods tend to offer a host of nutrients including fiber and plant-based sources of protein and have been recommended as important foods by the WHO to improve health and diet quality [107]. Potential reasons for the discrepant findings are multifactorial, although 1 potential reason could be related to supply chain issues which many countries experienced during the COVID-19 pandemic [14].

Other changes included most studies finding an increase in milk and milk products, snacking, and grain products. It is difficult to ascertain whether these increases are favorable, as, for example, milk and milk products include a host of products which could be regarded as “healthy” (e.g., yogurt and low-fat milk), whereas others might be regarded as foods to be limited (e.g., cheese and sugar-sweetened milk) [108]. Similarly, although grain products seem to have increased or remained stable during COVID-19, it is difficult to ascertain whether this is a positive change or not. Although most grain products (e.g., bread and pasta) provide important nutrients, there is a marked difference between refined grains and whole grains in terms of nutrient density, and by extension, health benefits [109], which most studies in this review did not differentiate between. Finally, although snacking increased, it is not inherently healthy or unhealthy [110], and is at least partially dependent upon the foods being consumed at these “snack” times, which was often not mentioned. Therefore, interpretation of the healthiness of these changes would be somewhat speculative.

This current review also found a similar number of studies suggesting either a decrease or no change in fish consumption during COVID-19, and a slight majority found an increase in meat, poultry, and eggs. Fish (specifically, fatty fish) are generally regarded as a healthy source of protein and the important fatty acids EPA and DHA, while also being a staple food within the Mediterranean Diet, which is often used as a measure of diet quality [111]. However, it should also be noted that fish can be a large source of toxins/contaminants, such as mercury and microplastics, which can be damaging to health [112]. Still, research has generally supported fatty fish’s positive contribution to health [113], and thus, if youth are consuming less, it may be detrimental to overall wellbeing should this trend continue beyond the COVID-19 pandemic. However, the finding that meat, poultry and eggs may have slightly increased could be regarded as either positive or negative. Although it is true that red meat has been labeled as a possible carcinogen (and 2/3 studies that investigated its intake found a significant increase), eggs and poultry conversely, have commonly been considered less detrimental to health, either offering neutral or positive health benefits [114,115]. As such, because of the limited number of studies which looked at these foods, and the somewhat mixed findings, it is unclear whether changes represent a positive or negative trend.

Similarly, there were mixed findings as to whether breakfast has increased or decreased during COVID-19 compared with prior. This is somewhat surprising, as evidence has suggested lack of time in the morning being a deterrent for consuming breakfast among youth [116], and by shifting to online education, it would be reasonable to assume there would be more time available during mornings, which could then lead to increased rates of breakfast consumption. However, because time is not the only deterrent to breakfast consumption, it is likely that other factors were involved as well, and there are other potential explanations such as youth not feeling pressured to eat in the mornings and having more flexibility in the time they choose to eat (compared with before the pandemic), which might explain why breakfast did not appear to increase during the COVID-19 pandemic. In addition, the finding that skipping breakfast and a general dysregulation of mealtimes among youth has been reported by a previous similar systematic review [102] further suggests that there are likely other factors involved.

When considering diet quality indices and overall assessments of diet quality, most studies found a positive change among youth, which is line with this review’s previous findings that consumption of ultraprocessed foods decreased, and fruit and vegetable consumption has increased. Although the concept of a “healthy diet” is somewhat subjective and certainly prone to social desirability bias, even when diet quality indices alone are considered, 2/3 studies found a significant increase in KIDMED score, and 1/1 found a significant increase in the Adolescent Food Habits Checklist Score, compared with one study which found dietary diversity scores were reduced in 2020, and the remainder of studies finding no significant differences in diet quality scores. Although these are a small number of studies utilizing diet quality indices, it does appear that overall diet quality seems to have increased during COVID-19, compared with prior.

Finally, as only 5 studies investigated nutrient intake, there is limited evidence to confidently assess whether substantial changes have been made, and if so, among which nutrients. For instance, whereas fat was the most studied nutrient (*n* = 4), only one study found a significant change during COVID-19, compared with prior. The remainder of the nutrients were largely not significantly different, which is somewhat surprising given that food intakes appeared to have changed. This may indicate that food changes were slight, and not large enough to alter overall nutrient intakes, or perhaps that changes in other food groups offset the overall nutrient intake changes that would have occurred. For example, in a study of both foods and nutrients, Lee et al. [68] found that in their study of 800 Korean teens, soft drink consumption was significantly increased during COVID-19 compared with prior, yet authors found no significant changes in sugar consumption (a notorious ingredient of soft drinks). In fact, the point estimate of sugar was found to decrease from 2019 to 2020, although this difference was not significant, suggesting that the increase in soft drink consumption may have been relatively small, or that other foods were changed to offset the impact on nutrient intake this might have been expected to have. It should also be noted that among the studies investigating nutrient intake, 3/5 would be considered “high quality” according to their study quality scores, and their rigor may have contributed to the null findings. Ultimately, however, more evidence is needed to definitively ascertain whether nutrient intake has significantly changed among youth during COVID-19, compared with prior, because of the limited number of studies investigating them.

This review is not without limitations. First, although most studies in this review used a statistical test to compare their sample’s findings to the overall population, there were a surprising number which did not, opting to present their data in the form of simple percentages and/or proportions. As such, making conclusive statements as to whether there were dietary changes within these studies is difficult, as some of these findings may be because of chance alone. However, to counteract the impact by which this might have on the findings, we have presented each increase as either a simple proportion change, or a statistically significant one, allowing for inferences to be made with this information. In addition, the findings of nonstatistically significant changes tended to mirror those of the statistically significant ones, demonstrating that although we may be less confident in those findings, they are at least not suggesting different conclusions.

Second, although we examined the quality of each study, each study was treated equally regarding analysis of food changes during the COVID-19 pandemic, and we opted to present a subanalysis of each dietary change, stratified by study quality instead (see Supplemental Table 3). This was done because studies which used a statistical test to report their findings were largely the same studies that were deemed higher quality (16/17 of “high quality” studies used a statistical test), and subgroup analysis suggested largely the same dietary changes among each group/habit. However, it should be noted that higher quality studies were much more likely to suggest null findings (particularly in the case of fruits and vegetables and ultraprocessed foods), which could potentially be attributed to their higher rigor. Still, because of the multitude of factors which determine the quality of a study within this review, and because the National Heart, Blood and Lung Institute’s Quality Assessment Tool for Observational Cohort and Cross-Sectional Studies [117] does not account for all these aspects, it was decided to use a more inclusive approach and not exclude any studies from the analysis portion. Finally, because experiencing a pandemic such as the COVID-19 pandemic is a rare phenomenon (i.e., 5 occurring in the past 100 y [118]), we felt it was best to gather as much information as possible, to gain a better understanding of dietary changes among youth, and not exclude any data.

Third, most studies in this review had an HDI score of “High” or “Very High” (63/67, 94.0%), which impacted our ability to assess whether findings were contingent on the level of development of the country under study. It also somewhat limits our ability to apply our findings and conclusions to countries with lower development indices who also dealt with dietary changes during the COVID-19 pandemic. It should also be noted that our study focused solely on youth, and not the family, which one review has done previously, noting the differences in dietary changes between families with children, and those without during the COVID-19 pandemic [119]. As such, both ideas represent an opportunity for future research to explore.

Lastly, this review did not attempt to quantify the magnitude of difference in food changes, which is especially important to consider when discussing food consumption changes. Unfortunately, because of the overwhelming number of ways that dietary changes were investigated, quantifying the differences across all studies would be problematic. This review found 268 unique measurements of food (see Supplemental Table 1) among the 67 studies, and even among those studying the same food, the frequency by which it was measured was often different between studies. This may be due in part to our search strategy and being somewhat unrestrictive of what could be considered a dietary change; however, we preferred this approach to consider a wider source of evidence. It is also noteworthy that most studies did not use the same questionnaires, meaning how the questions were asked also differed. As a result, although meta-analyses are useful for describing magnitudes of difference, we opted against attempting to do so.

In conclusion, results showed generally positive dietary changes among youth during COVID-19, including an increase in consumption of fruits and vegetables, decrease in intake of ultraprocessed foods, as well as a general increase in overall diet quality as measured both by diet quality indices and overall perception of changes. These are important to note as 2 previous reviews on adults have suggested negative findings regarding the impact of COVID-19 on diet quality [17,18]. Furthermore, we can speculate that although COVID-19 caused parents and their children to be at home more, it appears to have had a protective effect on diet quality. As such, to continue this protective effect, it is necessary to address the school food environment so children can have access to the same healthful foods offered at home, in hopes of maintaining the positive dietary changes made by youth during COVID-19. One way to do so is via a national school lunch program, and although most developed countries possess one, ensuring the healthiness of the meals being served is just as vital.

Findings for other food groups/habits were generally mixed, or interpretation of whether they were positive or not is somewhat unclear. This review provides evidence that although the pandemic has been associated with many negative health findings, most studies suggested positive, health-promoting changes with regards to food choices among youth. Future research should investigate whether changes made during the pandemic have persisted as restrictions continue to lift, and if so, what specific aspects of the COVID-19 pandemic have caused these food changes. Future research should also strongly consider using previously validated questionnaires to improve study quality and comparability between similar studies.

## Author contributions

The authors’ contributions were as follows – NW, JAS, JG: designed the research; NW, HS: conducted the research; JAG: provided essential reagents; NW, HS: analyzed data; NW, JAS, HS, SB, TT, JG: wrote the paper; NW: had primary responsibility for final content; and all authors: read and approved the final manuscript.

### Conflict of interest

The authors report no conflicts of interest.

## Funding

The authors reported no funding received for this study.

## Data availability

Any data used in this review that were not presented are available upon request.

## References

[1] WHO. WHO Director-General’s opening remarks at the media briefing on COVID-19—11 March 2020 [Internet]. [cited August 26, 2022]. Available from: https://www.who.int/director-general/speeches/detail/who-director-general-s-opening-remarks-at-the-media-briefing-on-covid-19---11-march-2020.

[2] Koh D. (2020). COVID-19 lockdowns throughout the world. Occup. Med. (Lond.)..

[3] Taeymans J., Luijckx E., Rogan S., Haas K., Baur H. (2021). Physical activity, nutritional habits, and sleeping behavior in students and employees of a Swiss university during the COVID-19 lockdown period: questionnaire survey study. JMIR Public Health Surveill.

[4] Czeisler M.É., Tynan M.A., Howard M.E., Honeycutt S., Fulmer E.B., Kidder D.P. (2020). Public attitudes, behaviors, and beliefs related to COVID-19, stay-at-home orders, nonessential business closures, and public health guidance—United States, New York City, and Los Angeles, May 5-12, 2020. MMWR Morb. Mortal. Wkly Rep..

[5] Bestetti R.B., Furlan-Daniel R., Couto L.B. (2022). Nonpharmaceutical public health interventions to curb the COVID-19 pandemic: a narrative review. J. Infect. Dev. Ctries..

[6] Camargo C.P., Tempski P.Z., Busnardo F.F., de Arruda Martins M., Gemperli R. (2020). Online learning and COVID-19: a meta-synthesis analysis. Clinics (Sao Paulo)..

[7] Jones E.A.K., Mitra A.K., Bhuiyan A.R. (2021). Impact of COVID-19 on mental health in adolescents: a systematic review. Int. J. Environ. Res. Public Health..

[8] Do B., Kirkland C., Besenyi G.M., Smock C., Lanza K. (2022). Youth physical activity and the COVID-19 pandemic: a systematic review. Prev. Med. Rep..

[9] Hensher M. (2020). COVID-19, unemployment, and health: time for deeper solutions?. BMJ.

[10] Idzerda L., Gariépy G., Corrin T., Tarasuk V., McIntyre L., Neil-Sztramko S. (2022). Evidence synthesis—what is known about the prevalence of household food insecurity in Canada during the COVID-19 pandemic: a systematic review. Health Promot. Chronic. Dis. Prev. Can..

[11] Adams E.L., Caccavale L.J., Smith D., Bean M.K. (2020). Food insecurity, the home food environment, and parent feeding practices in the era of COVID-19. Obesity (Silver Spring).

[12] Roberts A., Rogers J., Mason R., Siriwardena A.N., Hogue T., Whitley G.A. (2021). Alcohol and other substance use during the COVID-19 pandemic: a systematic review. Drug Alcohol. Depend..

[13] Lagström H., Stenholm S., Akbaraly T., Pentti J., Vahtera J., Kivimäki M. (2020). Diet quality as a predictor of cardiometabolic disease–free life expectancy: the Whitehall II cohort study. Am. J. Clin. Nutr..

[14] Aday S., Aday M.S. (2020). Impact of COVID-19 on the food supply chain. Food Qual. Saf..

[15] Panda P.K., Gupta J., Chowdhury S.R., Kumar R., Meena A.K., Madaan P. (2021). Psychological and behavioral impact of lockdown and quarantine measures for COVID-19 pandemic on children, adolescents and caregivers: a systematic review and meta-analysis. J. Trop. Pediatr..

[16] Nagata J.M., Abdel Magid H.S., Pettee Gabriel K. (2020). Screen time for children and adolescents during the coronavirus disease 2019 pandemic. Obesity (Silver Spring).

[17] Bennett G., Young E., Butler I., Coe S. (2021). The impact of lockdown during the COVID-19 outbreak on dietary habits in various population groups: a scoping review. Front. Nutr..

[18] González-Monroy C., Gómez-Gómez I., Olarte-Sánchez C.M., Motrico E. (2021). Eating behaviour changes during the COVID-19 pandemic: a systematic review of longitudinal studies. Int. J. Environ. Res. Public Health.

[19] Cost K.T., Crosbie J., Anagnostou E., Birken C.S., Charach A., Monga S. (2022). Mostly worse, occasionally better: impact of COVID-19 pandemic on the mental health of Canadian children and adolescents. Eur. Child Adolesc. Psychiatry..

[20] Lavelle F., Spence M., Hollywood L., McGowan L., Surgenor D., McCloat A. (2016). Learning cooking skills at different ages: a cross-sectional study. Int. J. Behav. Nutr. Phys. Activ..

[21] Seabrook J.A., Dworatzek P.D.N., Matthews J.I. (2019). Predictors of food skills in university students. Can. J. Dietet. Pract. Res..

[22] Srour B., Fezeu L.K., Kesse-Guyot E., Allès B., Méjean C., Andrianasolo R.M. (2019). Ultra-processed food intake and risk of cardiovascular disease: prospective cohort study (NutriNet-Santé). BMJ.

[23] Elizabeth L., Machado P., Zinöcker M., Baker P., Lawrence M. (2020). Ultra-processed foods and health outcomes: a narrative review. Nutrients.

[24] Pagliai G., Dinu M., Madarena M.P., Bonaccio M., Iacoviello L., Sofi F. (2021). Consumption of ultra-processed foods and health status: a systematic review and meta-analysis. Br. J. Nutr..

[25] Pourghazi F., Eslami M., Ehsani A., Ejtahed H.S., Qorbani M. (2022). Eating habits of children and adolescents during the COVID-19 era: a systematic review. Front. Nutr..

[26] Fanelli S.M., Jonnalagadda S.S., Pisegna J.L., Kelly O.J., Krok-Schoen J.L., Taylor C.A. (2020). Poorer diet quality observed among US adults with a greater number of clinical chronic disease risk factors. J. Prim. Care Community Health..

[27] Page M.J., McKenzie J.E., Bossuyt P.M., Boutron I., Hoffmann T.C., Mulrow C.D. (2021). The PRISMA 2020 statement: an updated guideline for reporting systematic reviews. BMJ.

[28] Veritas Health Innovation. Covidence systematic review software [Internet]. Melbourne, Australia [cited August 29, 2022]. Available from: https://www.covidence.org/.

[29] UNDP (United Nations Development Programme) (2022). Human Development Report 2021-22: Uncertain Times, Unsettled Lives: Shaping our Future in a Transforming World. New York.

[30] Health Canada (2019). https://food-guide.canada.ca/en/.

[31] Štefan L., Prosoli R., Juranko D., Čule M., Milinović I., Novak D. (2017). The reliability of the Mediterranean diet quality index (KIDMED) questionnaire. Nutrients.

[32] Aguilar-Martínez A., Bosque-Prous M., González-Casals H., Colillas-Malet E., Puigcorbé S., Esquius L. (2021). Social inequalities in changes in diet in adolescents during confinement due to covid-19 in spain: the deskcohort project. Nutrients.

[33] Al Hourani H., Alkhatib B., Abdullah M. (2022). Impact of COVID-19 lockdown on body weight, eating habits and physical activity of Jordanian children and adolescents. Disaster Med. Public Health Prep..

[34] Alfayez R.F., Albadr N.A., Abdelsamad A.I., Al-Masri S.A., Arzoo S., Alfayez M.F. (2022). COVID-19 pandemic and lifestyle changes: impact on school students. Prog Nutr.

[35] Almutairi N., Burns S., Portsmouth L. (2022). Nutritional behaviour of adolescents and the impact of COVID-19 on a diet in Saudi Arabia. Curr. Res. Nutr. Food Sci..

[36] Androutsos O., Perperidi M., Georgiou C., Chouliaras G. (2021). Lifestyle changes and determinants of children’s and adolescents’ body weight increase during the first COVID-19 lockdown in Greece: the COV-EAT study. Nutrients.

[37] Angoff H.D., Dial L.A., State F., Varga A.V., Kamath S., Musher-Eizenman D. (2022). Impact of stress and decision fatigue on parenting practices related to food and physical activity during COVID-19. Child Care Health Dev.

[38] Baghlaf K., Bormah D., Hakami A., Bagher S.M. (2022). The impact of the COVID-19 Lockdown on sugar-sweetened beverage consumption in children in Saudi Arabia: a mixed-methods study. Nutrients.

[39] Bahatheg R.O. (2021). Young Children’s nutrition during the COVID-19 pandemic lockdown: a comparative study. Early Child Educ. J..

[40] Bekelman T.A., Dong Y., Elliott A.J., Ferrara A., Friesen K., Galarce M. (2022). Health behavior changes during the COVID-19 pandemic: a longitudinal analysis among children. Int. J. Environ. Res. Public Health..

[41] Weihrauch-Blüher S., Huizinga O., Joisten C., Pflanz J., Torbahn G., Wiegand S. (2023). Changes in lifestyle and body weight in children and adolescents during the COVID-19 pandemic: a representative survey of parents in Germany. Obes. Facts..

[42] Borger C., Paolicelli C., Ritchie L., Whaley S.E., Dematteis J., Sun B. (2021). Shifts in sources of food but stable nutritional outcomes among children in the early months of the COVID-19 Pandemic. Int. J. Environ. Res. Public Health..

[43] Burkart S., Parker H., Weaver R.G., Beets M.W., Jones A., Adams E.L. (2022). Impact of the COVID-19 pandemic on elementary schoolers’ physical activity, sleep, screen time and diet: a quasi-experimental interrupted time series study. Pediatr. Obes..

[44] Calabriano V., Carrasco-Marín F., Ulloa N., Dávalos A., Ruiz-Roso M.B., Celis-Morales C. (2022). Modificación de estilos de vida de adolescentes chilenos durante el primer confinamiento por COVID-19. Rev. Med. Chil..

[45] Carroll N., Sadowski A., Laila A., Hruska V., Nixon M., Ma D.W.L. (2020). The impact of covid-19 on health behavior, stress, financial and food security among middle to high income Canadian families with young children. Nutrients.

[46] Cui Y., Si W., Zhao Q., Glauben T., Feng X. (2021). The impact of COVID-19 on the dietary diversity of children and adolescents: evidence from a rural/urban panel study. China World Econ.

[47] Díaz-Rodríguez M., Carretero-Bravo J., Pérez-Muñoz C., Deudero-Sánchez M. (2022). Lockdown due to COVID-19 in Spanish children up to 6 years: consequences on diet, lifestyle, screen viewing, and sleep. Int. J. Public Health..

[48] Dondi A., Candela E., Morigi F., Lenzi J., Pierantoni L., Lanari M. (2020). Parents’ perception of food insecurity and of its effects on their children in Italy six months after the COVID-19 pandemic outbreak. Nutrients.

[49] Dragun R., Veček N.N., Marendić M., Pribisalić A., Ðivić G., Cena H. (2020). Have lifestyle habits and psychological well-being changed among adolescents and medical students due to COVID-19 lockdown in Croatia?. Nutrients.

[50] Gardner L.A., Debenham J., Newton N.C., Chapman C., Wylie F.E., Osman B. (2022). Lifestyle risk behaviours among adolescents: a two-year longitudinal study of the impact of the COVID-19 pandemic. BMJ Open.

[51] Gedeon R., Hallit S., Wakim L.H. (2022). Food insecurity and eating habits of Lebanese children aged 5–11 years during the COVID-19 pandemic and the socioeconomic crisis: a national study. BMC Public Health.

[52] Hanbazaza M., Wazzan H. (2021). Changes in eating habits and lifestyle during COVID-19 curfew in children in Saudi Arabia. Nutr. Res. Pract..

[53] Hashem S.A., El Refay A.S., Mostafa H.H., Kamel I.H., Sherif L.S. (2020). Impact of coronavirus disease-19 lockdown on Egyptian children and adolescents: dietary pattern changes health risk, Open Access Maced. J. Med. Sci..

[54] He Y., Luo B., Zhao L., Liao S. (2022). Influences of the COVID-19 pandemic on obesity and weight-related behaviors among Chinese children: a multi-center longitudinal study. Nutrients.

[55] Horikawa C., Murayama N., Kojima Y., Tanaka H., Morisaki N. (2021). Changes in selected food groups consumption and quality of meals in Japanese school children during the COVID-19 pandemic. Nutrients.

[56] Husain Z., Ghosh S., Dutta M. (2022). Changes in dietary practices of mother and child during the COVID-19 lockdown: results from a household survey in Bihar, India. Food Policy.

[57] James M., Marchant E., Defeyter M.A., Woodside J., Brophy S. (2021). Impact of school closures on the health and well-being of primary school children in Wales, UK: a routine data linkage study using the HAPPEN Survey (2018–2020). BMJ Open.

[58] Jia P., Liu L., Xie X., Yuan C., Chen H., Guo B. (2021). Changes in dietary patterns among youths in China during COVID-19 epidemic: the COVID-19 impact on lifestyle change survey (COINLICS). Appetite.

[59] Kalyoncu I., Özcan G., Kargül B. (2021). Oral health practice and health-related quality of life of a group of children during the early stage of the COVID-19 pandemic in Istanbul. J. Educ. Health Promot..

[60] Kang S., Seo M.Y., Kim S.H., Park M.J. (2022). Changes in lifestyle and obesity during the COVID-19 pandemic in Korean adolescents: based on the Korea Youth Risk Behavior Survey 2019 and 2020. Ann. Pediatr. Endocrinol. Metab..

[61] Kim E. (2022). Alcohol drinking in adolescents due to the COVID-19 pandemic. Psychiatry Clin. Psychopharmacol..

[62] Kim S.Y., Yoo D.M., Min C., Choi H.G. (2021). Changes in dietary habits and exercise pattern of Korean adolescents from prior to during the COVID-19 pandemic. Nutrients.

[63] Kołota A., Głąbska D. (2021). COVID-19 pandemic and remote education contributes to improved nutritional behaviors and increased screen time in a polish population-based sample of primary school adolescents: diet and activity of youth during covid-19 (day-19) study. Nutrients.

[64] Kołota A., Głąbska D. (2021). Analysis of food habits during pandemic in a polish population-based sample of primary school adolescents: diet and activity of youth during COVID-19 (DAY-19) study. Nutrients.

[65] Konstantinou C., Andrianou X.D., Constantinou A., Perikkou A., Markidou E., Christophi C.A. (2021). Exposome changes in primary school children following the wide population non-pharmacological interventions implemented due to COVID-19 in Cyprus: a national survey. EClinicalMedicine.

[66] Kyan A., Takakura M. (2023). Impact of COVID-19 pandemic on the socioeconomic inequality of health behavior among Japanese adolescents: a two-year-repeated cross-sectional survey. J. Phys. Act Health.

[67] Lee J., Ko Y.H., Chi S., Lee M.S., Yoon H.K. (2022). Impact of the COVID-19 pandemic on Korean adolescents’ mental health and lifestyle factors. J. Adolescent Health.

[68] Lee H.A., Lee H.J., Park B., Shin Y., Park H., Park H. (2022). Changes in eating behaviors according to household income in adolescents during the COVID-19 pandemic: findings from the Korea National Health and Nutrition Examination Survey. Epidemiol. Health.

[69] López-Bueno R., López-Sánchez G.F., Casajús J.A., Calatayud J., Gil-Salmerón A., Grabovac I. (2020). Health-related behaviors among school-aged children and adolescents during the Spanish covid-19 confinement. Front. Pediatr..

[70] Luo M., Wang Q., Yang S., Jia P. (2022). Changes in patterns of take-away food ordering among youths before and after COVID-19 lockdown in China: the COVID-19 Impact on Lifestyle Change Survey (COINLICS). Eur. J. Nutr..

[71] Malta, D.C., Gomes, C.S., Barros, M.B.D.A., Lima, M.G., Silva, A.G.D., Cardoso, L.S.D.M., et al., (2021). The COVID-19 pandemic and changes in the lifestyles of Brazilian adolescents. *Revista Brasileira de Epidemiologia*, *24*, e210012. 10.1590/1980-549720210012 34105593

[72] Mastorci F., Piaggi P., Doveri C., Trivellini G., Casu A., Pozzi M. (2021). Health-related quality of life in Italian adolescents during COVID-19 outbreak. Front. Pediatr..

[73] Maximova K., Khan M.K.A., Dabravolskaj J., Maunula L., Ohinmaa A., Veugelers P.J. (2022). Perceived changes in lifestyle behaviours and in mental health and wellbeing of elementary school children during the first COVID-19 lockdown in Canada. Public Health.

[74] McNicholas J., Hammersley M.L., Hopkins S., McDermott S., Plaskett J. (2022). The impact of COVID-19 restrictions on the healthy eating and movement behaviors of 0–12-year-old children in Western Sydney, Australia. Front. Public Health.

[75] Medrano M., Cadenas-Sanchez C., Oses M., Arenaza L., Amasene M., Labayen I. (2021). Changes in lifestyle behaviours during the COVID-19 confinement in Spanish children: a longitudinal analysis from the MUGI project. Pediatr. Obes..

[76] Mikulec A., Zborowski M., Cisoń-Apanasewicz U., Stawiarska A., Kowalski S. (2022). The impact of the COVID-19 pandemic on the dietary habits of children and adolescents. Food Sci. Technol. Q..

[77] Moitra P., Madan J. (2022). Impact of screen time during COVID-19 on eating habits, physical activity, sleep, and depression symptoms: a cross-sectional study in Indian adolescents. PLOS ONE.

[78] Munasinghe S., Sperandei S., Freebairn L., Conroy E., Jani H., Marjanovic S. (2020). The impact of physical distancing policies during the COVID-19 pandemic on health and well-being among australian adolescents. J. Adolesc. Health.

[79] Nanayakkara J., Boddy G., Aydin G., Kombanda K.T., Larsson C., Worsley A. (2023). Australian parents’ and children’s food-related interactions during the COVID-19 pandemic. Br. Food J..

[80] Ng K., Cosma A., Svacina K., Boniel-Nissim M., Badura P. (2021). Czech adolescents’ remote school and health experiences during the spring 2020 COVID-19 lockdown. Prev. Med. Rep..

[81] Perrar I., Alexy U., Jankovic N. (2022). Changes in total energy, nutrients and food group intake among children and adolescents during the COVID-19 pandemic-results of the DONALD study. Nutrients.

[82] Philippe K., Chabanet C., Issanchou S., Monnery-Patris S. (2021). Child eating behaviors, parental feeding practices and food shopping motivations during the COVID-19 lockdown in France: (How) did they change?. Appetite.

[83] Pujia R., Ferro Y., Maurotti S., Khoory J., Gazzaruso C., Pujia A. (2021). The effects of COVID-19 on the eating habits of children and adolescents in Italy: a pilot survey study. Nutrients.

[84] Radwan A., Radwan E., Radwan W. (2021). Eating habits among primary and secondary school students in the Gaza Strip, Palestine: a cross-sectional study during the COVID-19 pandemic. Appetite.

[85] Ramos-Álvarez O., Arufe-Giráldez V., Cantarero-Prieto D., Ibáñez-García A. (2021). Impact of SARS-CoV-2 lockdown on anthropometric parameters in children 11/12 years old. Nutrients.

[86] Robinson L., Measey M.A., Efron D., Mundy L., Hoq M., Rhodes A. (2023). Effects of varying pandemic restrictions on the health-related behaviours of Australian children. J. Paediatr. Child Health..

[87] Rucińska M., Rutkowska N., Skowronek M., Matusik P., Zachurzok A. (2022). Desirable and undesirable lifestyle changes in polish children resulting from the COVID-19 pandemic. Polish J. Pediatr..

[88] Ruiz-Roso M.B., Padilha P de C., Mantilla-Escalante D.C., Ulloa N., Brun P., Acevedo-Correa D. (2020). COVID-19 confinement and changes of adolescent’s dietary trends in Italy, Spain, Chile, Colombia and Brazil. Nutrients.

[89] Saltaouras G., Perperidi M., Georgiou C., Androutsos O. (2022). Parental lifestyle changes and correlations with children’s dietary changes during the first COVID-19 lockdown in Greece: the COV-EAT study. Children.

[90] Schwarzová M., Fatrcová-Šramková K., Juríková T. (2022). Consequences of the COVID-19 pandemic on the eating habits of school-aged children in Slovakia. Journal of Hygienic Engineering and Design.

[91] Segre G., Campi R., Scarpellini F., Clavenna A., Zanetti M., Cartabia M. (2021). Interviewing children: the impact of the COVID-19 quarantine on children’s perceived psychological distress and changes in routine. BMC Pediatr.

[92] Shenoy S., Rao C., Somashekar A.R. (2021). Effect of lockdown during the novel coronavirus pandemic on diet and lifestyle of Indian children. Sri Lanka J. Child Health..

[93] Skolmowska D., Głąbska D., Guzek D. (2021). Differences in Adolescents’ Food Habits Checklist (AFHC) scores before and during pandemic in a population-based sample: Polish adolescents’ COVID-19 experience (place-19) study. Nutrients.

[94] Szczepańska E., Janota B. (2022). Lifestyle of families with children aged 4–8 years before and during lockdown due to COVID-19 pandemic in Poland. Int. J. Environ. Res. Public Health..

[95] Thiab S., Barakat M., Qudah R., Basheti I., Daoud S. (2022). Assessing health-related behaviors among Jordanian children during COVID-19 pandemic: a cross-sectional study. Pharm. Pract. (Granada)..

[96] van den Broek N., Larsen J.K., Verhagen M., Burk W.J., Vink J.M. (2023). Adolescents’ food intake changes during the COVID-19 pandemic: the moderating role of pre-pandemic susceptibility, COVID-19 related stressors, and the social food context. Eur. J. Dev. Psychol..

[97] Villodres G.C., García-Pérez L., Corpas J.M., Muros J.J. (2021). Influence of confinement due to COVID-19 on physical activity and Mediterranean diet adherence and its relationship with self-esteem in pre-adolescent students. Children (Basel).

[98] Wang D., Chukwu A., Millogo O., Assefa N., James C., Young T. (2021). The COVID-19 pandemic and adolescents’ experience in sub-Saharan Africa: a cross-country study using a telephone survey. Am. J. Trop. Med. Hyg..

[99] De Oliveira Figueiredo R.A., Viljakainen J., Viljakainen H., Roos E., Rounge T.B., Weiderpass E. (2019). Identifying eating habits in Finnish children: a cross-sectional study. BMC Public Health.

[100] Lange S.J., Moore L.V., Harris D.M., Merlo C.L., Seung H.L. (2017). https://www.cdc.gov/mmwr/mmwr_continuingEducation.html.

[101] Minaker L., Hammond D. (2016). Low frequency of fruit and vegetable consumption among Canadian youth: findings from the 2012/2013 youth smoking survey. J. Sch. Health..

[102] Karatzi K., Poulia K.A., Papakonstantinou E., Zampelas A. (2021). The impact of nutritional and lifestyle changes on body weight, body composition and cardiometabolic risk factors in children and adolescents during the pandemic of COVID-19: a systematic review. Children (Basel)..

[103] Sarda B., Delamaire C., Serry A.J., Ducrot P. (2022). Changes in home cooking and culinary practices among the French population during the COVID-19 lockdown. Appetite.

[104] Maclellan D.L., Gottschall-Pass K., Larsen R. (2007). Fruit and vegetable consumption: benefits and barriers. Can. J. Diet. Pract. Res..

[105] Kipnis V., Midthune D., Freedman L., Bingham S., Day N.E., Riboli E. (2002). Bias in dietary-report instruments and its implications for nutritional epidemiology. Public Health Nutr.

[106] Hebert J.R., Clemow L., Pbert L., Ockene I.S., Ockene J.K. (1995). Social desirability bias in dietary self-report may compromise the validity of dietary intake measures. Int. J. Epidemiol..

[107] (2021). Plant-based diets and their impact on health, sustainability and the environment: a review of the evidence: WHO European Office for the Prevention and Control of Noncommunicable Diseases.

[108] Eat protein foods—Canada’s food guide [Internet] [cited August 26, 2022]. Available from: https://food-guide.canada.ca/en/healthy-eating-recommendations/make-it-a-habit-to-eat-vegetables-fruit-whole-grains-and-protein-foods/eat-protein-foods/.

[109] Gaesser G.A. (2020). Whole grains, refined grains, and cancer risk: a systematic review of meta-analyses of observational studies. Nutrients.

[110] Marangoni F., Martini D., Scaglioni S., Sculati M., Donini L.M., Leonardi F. (2019). Snacking in nutrition and health. Int. J. Food Sci. Nutr..

[111] Huhn S., Masouleh S.K., Stumvol M., Villringer A., Witte A.V. (2015). Components of a Mediterranean diet and their impact on cognitive functions in aging. Front. Aging Neurosci..

[112] Landrigan P.J., Stegeman J.J., Fleming L.E., Allemand D., Anderson D.M., Backer L.C. (2020). Human health and ocean pollution. Ann. Glob. Health..

[113] Giosuè A., Calabrese I., Lupoli R., Riccardi G., Vaccaro O., Vitale M. (2022). Relations between the consumption of fatty or lean fish and risk of cardiovascular disease and all-cause mortality: a systematic review and meta-analysis. Adv. Nutr..

[114] Lichtenstein A.H., Appel L.J., Vadiveloo M., Hu F.B., Kris-Etherton P.M., Rebholz C.M. (2021). Dietary guidance to improve cardiovascular health: a scientific statement from the American Heart Association. Circulation.

[115] Drouin-Chartier J.P., Chen S., Li Y., Schwab A.L., Stampfer M.J., Sacks F.M. (2020). Egg consumption and risk of cardiovascular disease: three large prospective US cohort studies, systematic review, and updated meta-analysis. BMJ.

[116] Hearst M.O., Shanafelt A., Wang Q., Leduc R., Nanney M.S. (2016). Barriers, benefits, and behaviors related to breakfast consumption among rural adolescents. J. Sch. Health..

[117] Study Quality Assessment Tools. NHLBI, NIH [cited August 26, 2022]. Available from: https://www.nhlbi.nih.gov/health-topics/study-quality-assessment-tools.

[118] Poorolajal J. (2021). The global pandemics are getting more frequent and severe. J. Res. Health Sci..

[119] Campbell H., Wood A.C. (2021). Challenges in feeding children posed by the COVID-19 pandemic: a systematic review of changes in dietary intake combined with a dietitian’s perspective. Curr. Nutr. Rep..

