# Supplementary material for: Dietary Changes of Youth during the COVID-19 Pandemic: A Systematic Review
Source: J Nutr. 2024 Feb 24;154(4):1376–403. doi: 10.1016/j.tjnut.2024.02.022 (PMC11007747; doi:10.1016/j.tjnut.2024.02.022)
Supplement: Multimedia component 1 [file mmc1.docx]

**Supplementary Table 1: Search Strategy:**

| **MEDLINE/EMBASE (Ovid)** | **CINAHL** | **Scopus** |
| --- | --- | --- |
| “(food craving/ or food quantity/ or food/ or "takeaway (food)"/ or food intake/ or food frequency questionnaire/ or minimally processed food/ or processed food/ or ultra-processed food/ or diet composition/ or Mediterranean diet/ or unhealthy diet/ or diet/ or healthy diet/ or high calorie diet/ or Western diet/ or nutrient intake/ or nutrient/ or meal skipping/ or meal frequency/ or meal/ or fast food/ or dietary intake/ or diet/ or cooking/ or fruit consumption/ or fruit/ or vegetable consumption/ or vegetable/ or diet$.mp. or fast food$.mp. or food$.mp.) AND (child/ or adolescent/ or juvenile/ or youth.mp. or teen$.mp. or pediatrics/) AND limit to (yr="2020 -Current" and covid-19)”. | “(MH "Food") OR (MH "Food Preferences") OR (MH "Food Habits") OR (MH "Food Intake") OR (MH "Food and Beverages") OR (MH "Portion Size") OR (MH "Diet Records") OR (MH "Diet Fads") OR (MH "Snacks") OR (MH "Diet") OR (MH "Mediterranean Diet") OR (MH "Diet, Western") OR (MH "Meal Preparation") OR (MH "Meals") OR (MH "Snacks") OR (MH "Cooking") OR (MH "Fruit") OR (MH "Vegetables") OR diet* OR "fast food*" OR food*” AND “(MH "Child") OR (MH "Child Behavior") OR (MH "Child Health") OR (MH "Child Nutrition") OR (MH "Pediatric Obesity") OR (MH "Adolescence") OR teen* OR youth* OR child*” AND “(MH "COVID-19") OR (MH "SARS-CoV-2") OR "covid-19" OR "corona*"” with a published date limited to January 1^st^ 2020 – May 1^st^ 2023. | “TITLE-ABS-KEY ( ( teen* OR adolescent* OR youth* OR child* ) AND ( covid* OR corona* OR pandemic OR epidemic ) AND ( food* OR nutrient* OR diet* OR meal* ) ) AND ( LIMIT-TO ( PUBYEAR , 2023 ) OR LIMIT-TO ( PUBYEAR , 2022 ) OR LIMIT-TO ( PUBYEAR , 2021 ) OR LIMIT-TO ( PUBYEAR , 2020 ) )” |

| **Supplementary Table 2: Categorization of Food Measures** | | |
| --- | --- | --- |
| **New Dietary Change Group** | **Foods Included** | **Number of Times Each Dietary Change was Studied** |
| **Fruits and Vegetables** | Fruits | 30 |
|  | Vegetables | 24 |
|  | Fruits and Vegetables | 11 |
|  | Fresh Fruit | 3 |
|  | Fruit Juice | 3 |
|  | Fresh Vegetables | 2 |
|  | Vegetables/Salad | 2 |
|  | 100% Fruit Juice | 1 |
|  | At Least One Serving of Vegetables (excluding Potatoes) or Salad with Evening Meal | 1 |
|  | Canned Juice | 1 |
|  | Cooked Vegetables | 1 |
|  | Dry Fruit | 1 |
|  | Fresh and Dried Fruits | 1 |
|  | Freshly prepared fruit juices | 1 |
|  | Fruit and Vegetable Juices | 1 |
|  | Fruit Fresh | 1 |
|  | Fruits and Fresh Juices | 1 |
|  | Fruits that are yellow or orange inside | 1 |
|  | Green Leafy Vegetables | 1 |
|  | Healthy Food (Fruits and Vegetables) | 1 |
|  | Juice | 1 |
|  | Other Fruits and Vegetables | 1 |
|  | Packaged 100% Fruit Juices | 1 |
|  | Potato | 1 |
|  | Preserved Vegetables | 1 |
|  | Raw Vegetables | 1 |
|  | Tubers | 1 |
|  | Vegetable Fresh | 1 |
|  | Vegetables (green leafy vegetables, orange and yellow vegetables, salad, and other vegetables) | 1 |
|  | Vegetables Dishes or Salad | 1 |
|  | Whole Fruit | 1 |
| **Fish and Aquatic Products** | Fish | 8 |
|  | Aquatic Products | 1 |
| **Grain Products** | Bread | 3 |
|  | Pasta and Rice | 2 |
|  | Rice | 2 |
|  | Bread and/or cereal (e..g 1 slice of bread, ½ bread roll, ½ cup breakfast cereal, or ½ cup rice, pasta, or noodles ) | 1 |
|  | Cereal Preparations | 1 |
|  | Cereal products | 1 |
|  | Cereals | 1 |
|  | Cereals for Breakfast | 1 |
|  | Cereals, Cereal Bars | 1 |
|  | Cornflakes | 1 |
|  | Grains | 1 |
|  | Macaroni and Pasta | 1 |
|  | Pasta | 1 |
|  | Refined Grains | 1 |
|  | Wheat Bread | 1 |
|  | Wheat Products | 1 |
|  | Whole Grains | 1 |
|  | Whole Wheat Bread | 1 |
| **Legumes, Beans, Seeds, and Nuts** | Legumes | 8 |
|  | Nuts | 3 |
|  | Pulses | 2 |
|  | Nuts, Seeds | 1 |
|  | Pulses and Nuts | 1 |
|  | Soybean Products | 1 |
|  | Soybean Products and Nuts | 1 |
| **Meat, Poultry, and Eggs** | Eggs | 5 |
|  | Meat | 4 |
|  | Poultry | 3 |
|  | Red Meat | 3 |
|  | Boiled Eggs | 1 |
|  | Chicken | 1 |
|  | Meat and meat products | 1 |
| **Milk and Milk Products** | Dairy | 5 |
|  | Milk | 4 |
|  | Milk and dairy products | 3 |
|  | Cheese | 2 |
|  | Dairy Products | 2 |
|  | Cheeses and cottage cheese | 1 |
|  | Creamy Cheese | 1 |
|  | Dairy for Breakfast | 1 |
|  | Labaneh | 1 |
|  | Milk and Cheese | 1 |
|  | Milk and Milk Products | 1 |
|  | Milk, Cheese, and Yogurt | 1 |
|  | Yoghurt, Cheese, Quark | 1 |
|  | Yoghurts and fermented milk drinks | 1 |
| **Breakfast** | Breakfast | 13 |
| **Nutrients** | Fat | 4 |
|  | Calories | 3 |
|  | Sugar | 3 |
|  | Calcium | 2 |
|  | Sodium | 2 |
|  | Added Sugars | 1 |
|  | Carbohydrate | 1 |
|  | Fatty Acid Ratio | 1 |
|  | Fiber | 1 |
|  | Free Sugar | 1 |
|  | Potassium | 1 |
|  | Protein | 1 |
|  | Saturated Fat | 1 |
|  | Vitamin A | 1 |
|  | Vitamin D | 1 |
| **Ultra-Processed Foods** | Fast-Food | 15 |
|  | Soft Drinks | 9 |
|  | Sugar-Sweetened Beverages | 8 |
|  | Sweet Snacks | 4 |
|  | Sweets | 4 |
|  | Carbonated Beverages | 3 |
|  | Fried Food | 3 |
|  | Unhealthy food | 3 |
|  | Candies | 2 |
|  | Dessert/Pudding | 2 |
|  | Discretionary food | 2 |
|  | Ice Cream | 2 |
|  | Packaged Food | 2 |
|  | Potato Chips | 2 |
|  | Processed Meat | 2 |
|  | Sausages/Burgers | 2 |
|  | Soda | 2 |
|  | Sugary Drinks | 2 |
|  | Takeaway Meals | 2 |
|  | Ultra-Processed Food | 2 |
|  | Arabic Sweets | 1 |
|  | Cake | 1 |
|  | Cake and sweets | 1 |
|  | Cakes and Pastries | 1 |
|  | Cakes, Biscuits and Cupcakes | 1 |
|  | Candy | 1 |
|  | Candy, Chocolate | 1 |
|  | Chips | 1 |
|  | Chips, Salty Biscuits | 1 |
|  | Chocolate | 1 |
|  | Chocolate Bar | 1 |
|  | Chocolates and Sweets | 1 |
|  | Chocolates or Sweets | 1 |
|  | Coffee and Caffeinated Drinks | 1 |
|  | Compote, Fruits in Syrup | 1 |
|  | Convenience Food | 1 |
|  | Cream Dessert | 1 |
|  | Dessert | 1 |
|  | Desserts (not low-fat) and Other Sweets | 1 |
|  | Eating Out | 1 |
|  | Eating out with family and/or friends | 1 |
|  | Energy Drinks | 1 |
|  | Fast food products (kebab, pizza, hamburger) | 1 |
|  | Fast Food/Takeout | 1 |
|  | Fast-Food Meals or Snacks | 1 |
|  | Fizzy Drinks | 1 |
|  | Flavoured milk | 1 |
|  | Food Ordering | 1 |
|  | French Fried Potatoes | 1 |
|  | French Fries | 1 |
|  | Fries | 1 |
|  | Frozen Food (pizza, nuggets, pies) | 1 |
|  | Frozen foods (e.g. pizza, lasagna or other frozen ready dish) | 1 |
|  | Honey | 1 |
|  | Ice Cream and Desserts | 1 |
|  | Jams | 1 |
|  | Junk Food | 1 |
|  | Junk Food and Sweets | 1 |
|  | Junk food/fast food and fried food | 1 |
|  | Muesli and breakfast cereals - sweet | 1 |
|  | Ordering takeaways | 1 |
|  | Packaged snacks (e.g. granola bars, chips) | 1 |
|  | Pastries (Fatayer) | 1 |
|  | Pastries for Breakfast | 1 |
|  | Pastries, Cake, Sweet Cookies | 1 |
|  | Pizza | 1 |
|  | Ready-Made Food | 1 |
|  | Regular (not low-fat) Snack Chips or Crackers | 1 |
|  | Restaurant, Takeaway, and Delivery | 1 |
|  | Salty/Savoury Snacks | 1 |
|  | Sandwich, Pizza, Savory Pies | 1 |
|  | Snacks (Junk Food) | 1 |
|  | Snacks, fast food, and high-calorie foods | 1 |
|  | Soda and energy drinks | 1 |
|  | Sodas or Glasses of Sweet Tea | 1 |
|  | Soft drink/energy/sport/fruit drink | 1 |
|  | sugar-sweetened beverages (carbonated soft drinks, sugar-sweetened juices) | 1 |
|  | Sugary Snack | 1 |
|  | Sweet Beverages | 1 |
|  | Sweet Drinks | 1 |
|  | Sweet Food | 1 |
|  | Sweet Packaged Snacks | 1 |
|  | Sweet/Candy/Chips | 1 |
|  | Sweetened drinks (coca cola, flavored waters) | 1 |
|  | Sweetened Juices | 1 |
|  | Sweets (cakes, cookies, candies) | 1 |
|  | Sweets and Jelly Beans | 1 |
|  | Sweets and Pastries | 1 |
|  | Sweets and Snacks | 1 |
|  | Sweets and Unhealthy Food | 1 |
|  | Sweets/candies/chocolates | 1 |
|  | Takeaway | 1 |
|  | Unhealthy food when he/she is bored or stressed or upset | 1 |
|  | Unhealthy foods (potato chips, chocolate biscuits and cake, and soft drink, cordial or juice) | 1 |
|  | Unhealthy meals (fast food, sweets, fizzy drinks, chips…etc.) | 1 |
|  | Unhealthy Snacks (e.g. a chocolate bar, a piece of cake, a packet of chips /corn chips, ice cream, 3-4 sweet biscuits) | 1 |
|  | unhealthy snacks (foods high in fat, salt, and sugar) | 1 |
|  | White bread and pastry | 1 |
| **Snacking** | Salty Snacks | 3 |
|  | Savory Snacks | 2 |
|  | Snacks | 2 |
|  | Snacks Between Meals | 2 |
|  | Late Snacks During Night | 1 |
|  | Number of Snacks | 1 |
|  | Salty Snacks (chips, peanuts, sticks) | 1 |
|  | Snack Foods | 1 |
|  | Snacks after dinner | 1 |
|  | Total Snacks | 1 |
| **Diet Quality Indices and Overall Assessments** | Total KIDMED Score | 3 |
|  | Healthy Diet | 2 |
|  | AFHC Consumption Score | 1 |
|  | Balanced Diet (including health ingredients such as whole wheat, pulses, legumes, eggs, nuts, fruits and vegetables) | 1 |
|  | Dietary Diversity Score | 1 |
|  | Dietary Score | 1 |
|  | Eating Habits | 1 |
|  | Eating Less Healthy | 1 |
|  | Eating More Healthy | 1 |
|  | Healthy Eating Index-2015 | 1 |
| **No Applicable Food Group** | Number of meals | 6 |
|  | Quantity of Food | 4 |
|  | Tea | 3 |
|  | Coffee | 2 |
|  | Lunch | 2 |
|  | Meat and cold cuts | 2 |
|  | Oil, Butter, and Margarine | 2 |
|  | Amount of Food | 1 |
|  | Animal-Source Foods | 1 |
|  | Beans, Chicken, or Fish | 1 |
|  | Bread and pastry - wholemeal | 1 |
|  | Bread, Pizza, and Bakery Products | 1 |
|  | Cereals and potatoes | 1 |
|  | Dinner | 1 |
|  | Eating as Normal | 1 |
|  | Eating Same Quality of Food | 1 |
|  | Fish and meat | 1 |
|  | Food Consumption | 1 |
|  | Food Intake | 1 |
|  | Food Items that Contain Sugar | 1 |
|  | Fresh Food | 1 |
|  | Greens and Beans | 1 |
|  | Healthy Food | 1 |
|  | Healthy food like fruit, vegetables, meat and milk | 1 |
|  | Healthy meals (balanced meals including fruit, vegetables, protein…etc.) | 1 |
|  | High Saturated Fat Food | 1 |
|  | Immunity-boosting foods (lemon, turmeric, garlic, citrus fruits and green leafy vegetables) | 1 |
|  | Impulsive eating | 1 |
|  | Less Food | 1 |
|  | Lunch or dinner with family | 1 |
|  | Margarine, Butter, or Meat Fat to Season Vegetables, or put on Potatoes, Bread, or Corn | 1 |
|  | Meals containing fresh foods | 1 |
|  | Meat and Processed Meat | 1 |
|  | Meat, Fish, and Eggs | 1 |
|  | Meat, Fish, or Eggs | 1 |
|  | More Food | 1 |
|  | Nutrition supplements to boost immunity | 1 |
|  | Olive Oil | 1 |
|  | Other Beverages | 1 |
|  | Other Staple Foods | 1 |
|  | Perceived unhealthiness | 1 |
|  | Popcorn | 1 |
|  | Potatoes, pasta, rice | 1 |
|  | Prepacked Juices and Sodas | 1 |
|  | Protein Foods | 1 |
|  | Quantity and Quality of Food | 1 |
|  | Quantity/portions of meals and snacks | 1 |
|  | Seafood and Plant Protein | 1 |
|  | Skipping one of the main meals (breakfast/lunch/dinner) | 1 |
|  | Staples | 1 |
|  | Supplements | 1 |
|  | Support in eating healthy | 1 |
|  | Variety of Foods | 1 |
|  | Vegetables, Legumes and Fruits | 1 |
|  | Watching television while having meals | 1 |
|  | Water | 1 |
