# Supplementary material for: Dietary Changes of Youth during the COVID-19 Pandemic: A Systematic Review
Source: J Nutr. 2024 Feb 24;154(4):1376–403. doi: 10.1016/j.tjnut.2024.02.022 (PMC11007747; doi:10.1016/j.tjnut.2024.02.022)
Supplement: Multimedia component 2 [file mmc2.docx]

**Supplementary Table 3: Dietary Change Findings, by Study Quality**

| **Fruits and Vegetables (n=46)** | | |
| --- | --- | --- |
| **Quality: Good (≥6)** | | |
| ***Author*** | **Quality Rating** | **Findings** |
| *Perrar et al.* | 9 | No change in consumption |
| *Medrano et al.* | 8 | No change in consumption |
| *Ramos-Álvarez et al.* | 8 | Increased consumption |
| *Szczepanska et al.* | 7 | No change in consumption |
| *Borger et al.* | 6 | Increased consumption |
| *Cui et al.* | 6 | Decreased consumption |
| *Gardner et al.* | 6 | Decreased consumption |
| *Husain et al.* | 6 | Increased consumption |
| *Kim et al.* | 6 | No change in consumption |
| *Kołota et al.* | 6 | Increased consumption |
| *Lee H.A. et al* | 6 | Decreased consumption |
| *Munasinghe et al.* | 6 | No change in consumption |
| *Van den Broek et al.* | 6 | Decreased consumption |
| ***Overall: 4 Increased, 4 Decreased, 5 No Change*** | | |
| **Quality: Moderate (4-5)** | | |
| *Aguilar-Martínez et al.* | 5 | Increased consumption |
| *Dragun et al.* | 5 | Increased consumption |
| *Gedeon et al.* | 5 | Decreased consumption |
| *James et al.* | 5 | No change in consumption |
| *Kang et al.* | 5 | Decreased consumption |
| *Kołota et al.* | 5 | Increased consumption |
| *Moitra et al.* | 5 | Increased consumption |
| *Baghlaf et al.* | 4 | Decreased consumption |
| *Carroll et al.* | 4 | Increased consumption |
| *Hanbazaza et al.* | 4 | No change in consumption |
| *Jia et al.* | 4 | Decreased consumption |
| *Kalyoncu et al.* | 4 | Increased consumption |
| *Philippe et al.* | 4 | Increased consumption |
| *Robinson et al.* | 4 | Increased consumption |
| *Ruiz-Roso et al.* | 4 | Increased consumption |
| *Skolmowska et al.* | 4 | Increased consumption |
| ***Overall: 10 Increased, 4 Decreased, 2 No Change*** | | |
| **Quality: Low (≤3)** | | |
| *Al Hourani et al.* | 3 | Increased consumption |
| *Alfayez et al.* | 3 | Increased consumption |
| *Horikawa et al.* | 3 | Decreased consumption |
| *Konstantinou et al.* | 3 | No change in consumption |
| *López-Bueno et al.* | 3 | Decreased consumption |
| *McNicholas et al.* | 3 | Increased consumption |
| *Ng et al.* | 3 | Increased consumption |
| *Pujia et al.* | 3 | Increased consumption |
| *Bahatheg* | 2 | Unclear |
| *Calabriano et al.* | 2 | Decreased consumption |
| *Radwan et al.* | 2 | Increased and decreased consumption |
| *Wang et al.* | 2 | Decreased consumption |
| *Androutsos et al.* | 1 | Increased consumption |
| *Dondi et al.* | 1 | Unclear |
| *Malta et al.* | 1 | Increased consumption |
| *Schwarzova et al.* | 1 | Increased consumption |
| *Shenoy et al.* | 1 | Increased consumption |
| ***Overall: 10 Increased, 5 Decreased, 1 No Change, 2 Unclear*** | | |

| **Ultra-Processed Foods (n=53)** | | |
| --- | --- | --- |
| **Quality: Good (≥6)** | | |
| ***Author*** | **Quality Rating** | **Findings** |
| *Perrar et al.* | 9 | No change in consumption |
| *Burkart et al.* | 8 | Increased consumption |
| *Medrano et al.* | 8 | No change in consumption |
| *Ramos-Álvarez et al.* | 8 | No change in consumption |
| *He et al.* | 7 | Decreased consumption |
| *Lee H.A. et al.* | 7 | Increased and decreased consumption |
| *Bekelman et al.* | 6 | No change in consumption |
| *Gardner et al.* | 6 | Decreased consumption |
| *Kim et al.* | 6 | Decreased consumption |
| *Kołota et al.* | 6 | No change in consumption |
| *Munasinghe et al.* | 6 | Decreased consumption |
| *Szczepanska et al.* | 6 | Increased and decreased consumption |
| *Van den Broek et al.* | 6 | Decreased consumption |
| ***Overall: 3 Increased, 7 Decreased, 5 No Change*** | | |
| ***Quality: Moderate (4-5)*** | | |
| *Aguilar-Martínez et al.* | 5 | Decreased consumption |
| *Dragun et al.* | 5 | Decreased consumption |
| *Gedeon et al.* | 5 | Increased and decreased consumption |
| *James et al.* | 5 | Increased and decreased consumption |
| *Kang et al.* | 5 | No change in consumption |
| *Kołota et al.* | 5 | No change in consumption |
| *Luo et al.* | 5 | Decreased consumption |
| *Moitra et al.* | 5 | Increased consumption |
| *Baghlaf et al.* | 4 | Decreased consumption |
| *Carroll et al.* | 4 | Decreased consumption |
| *Hanbazaza et al.* | 4 | Decreased consumption |
| *Jia et al.* | 4 | Decreased consumption |
| *Kalyoncu et al.* | 4 | Decreased consumption |
| *Philippe et al.* | 4 | Increased and decreased consumption |
| *Robinson et al.* | 4 | Increased consumption |
| *Ruiz-Roso et al.* | 4 | Increased and decreased consumption |
| *Skolmowska et al.* | 4 | Decreased consumption |
| ***Overall: 6 Increased, 13 Decreased, 2 No Change*** | | |
| ***Quality: Low (≤3)*** | | |
| *Al Hourani et al.* | 3 | Increased consumption |
| *Alfayez et al.* | 3 | Increased consumption |
| *Almutairi et al.* | 3 | Decreased consumption |
| *Konstantinou et al.* | 3 | Decreased consumption |
| *McNicholas et al.* | 3 | Increased consumption |
| *Ng et al.* | 3 | Increased and decreased consumption |
| *Pujia et al.* | 3 | Increased and decreased consumption |
| *Rucinska et al.* | 3 | Increased and decreased consumption |
| *Segre et al.* | 3 | Increased consumption |
| *Bahatheg* | 2 | Unclear |
| *Calabriano et al.* | 2 | Decreased consumption |
| *Diaz-Rodriguez et al.* | 2 | Increased consumption |
| *Radwan et al.* | 2 | Decreased consumption |
| *Saltaouras et al.* | 2 | Decreased consumption |
| *Thiab et al.* | 2 | Increased consumption |
| *Androutsos et al.* | 1 | Increased and decreased consumption |
| *Angoff et al.* | 1 | Decreased consumption |
| *Dondi et al.* | 1 | Unclear |
| *Hashem et al.* | 1 | Unclear |
| *Malta et al.* | 1 | Increased and decreased consumption |
| *Mikulec et al.* | 1 | Increased consumption |
| *Schwarzova et al.* | 1 | Increased consumption |
| *Shenoy et al.* | 1 | Decreased consumption |
| ***Overall: 13 Increased, 12 Decreased, 3 Unclear*** | | |

| **Milk and Milk Products (n=15)** | | |
| --- | --- | --- |
| **Quality: Good (≥6)** | | |
| ***Author*** | **Quality Rating** | **Findings** |
| *Medrano et al.* | 8 | No change in consumption |
| *Ramos-Álvarez et al.* | 8 | Decreased consumption |
| *Borger et al.* | 6 | No change in consumption |
| *Cui et al.* | 6 | Increased consumption |
| *Husain et al.* | 6 | Decreased consumption |
| ***Overall: 1 Increased, 2 Decreased, 2 No Change*** | | |
| ***Quality: Moderate (4-5)*** | | |
| *Aguilar-Martínez et al.* | 5 | Increased consumption |
| *Hanbazaza et al.* | 4 | Decreased consumption |
| *Jia et al.* | 4 | Decreased consumption |
| *Philippe et al.* | 4 | Increased consumption |
| ***Overall: 2 Increased, 2 Decreased*** | | |
| **Quality: Low (≤3)** | | |
| *Al Hourani et al.* | 3 | Increased consumption |
| *Horikawa et al.* | 3 | Decreased consumption |
| *Pujia et al.* | 3 | Increased consumption |
| *Bahatheg* | 2 | Unclear |
| *Androutsos et al.* | 1 | Increased consumption |
| *Schwarzova et al.* | 1 | Increased consumption |
| ***Overall: 4 Increased, 1 Decreased, 1 Unclear*** | | |

| **Legumes, Beans, Seeds, and Nuts (n=13)** | | |
| --- | --- | --- |
| **Quality: Good (≥6)** | | |
| ***Author*** | **Quality Rating** | **Findings** |
| *Medrano et al.* | 8 | Increased consumption |
| *Ramos-Álvarez et al.* | 8 | No change in consumption |
| *Cui et al.* | 6 | Decreased consumption |
| *Husain et al.* | 6 | Increased consumption |
| ***Overall: 2 Increased, 1 Decreased, 1 No Change*** | | |
| **Quality: Moderate (4-5)** | | |
| *Aguilar-Martínez et al.* | 5 | Decreased consumption |
| *Jia et al.* | 4 | Decreased consumption |
| *Philippe et al.* | 4 | Increased consumption |
| *Ruiz-Roso et al.* | 4 | Increased consumption |
| ***Overall: 2 Increased, 2 Decreased*** | | |
| ***Quality: Low (≤3)*** | | |
| *Konstantinou et al.* | 3 | No change in consumption |
| *Calabriano et al.* | 2 | *Decreased consumption* |
| *Wang et al.* | 2 | Decreased consumption |
| *Androutsos et al.* | 1 | No change in consumption |
| *Schwarzova et al.* | 1 | *No change in consumption* |
| ***Overall: 2 Decreased, 3 No Change*** | | |

| **Grain Products (n=11)** | | |
| --- | --- | --- |
| **Quality: Good (≥6)** | | |
| ***Author*** | **Quality Rating** | **Findings** |
| *Medrano et al.* | 8 | No change in consumption |
| *Ramos-Álvarez et al.* | 8 | No change in consumption |
| *Borger et al.* | 6 | Increased consumption |
| *Cui et al.* | 6 | No change in consumption |
| ***Overall: 1 Increased, 3 No Change*** | | |
| ***Quality: Moderate (4-5)*** | | |
| *Aguilar-Martínez et al.* | 5 | Increased consumption |
| *Jia et al.* | 4 | Increased and decreased consumption |
| *Philippe et al.* | 4 | No change in consumption |
| ***Overall: 2 Increased, 1 Decreased, 1 No Change*** | | |
| ***Quality: Low (≤3)*** | | |
| *Al Hourani et al.* | 3 | Increased consumption |
| *Pujia et al.* | 3 | Increased consumption |
| *Androutsos et al.* | 1 | Increased consumption |
| Schwarzova et al. | 1 | No change in consumption |
| ***Overall: 3 Increased, 1 No Change*** | | |

| **Fish and Aquatic Products (n = 9)** | | |
| --- | --- | --- |
| **Quality: Good (≥6)** | | |
| **Author** | **Quality Rating** | **Findings** |
| *Ramos-Álvarez et al.* | 8 | No change in consumption |
| *Medrano et al.* | 8 | No change in consumption |
| *Cui et al.* | 6 | Decreased consumption |
| ***Overall: 1 Decreased, 2 No Change*** | | |
| **Quality: Moderate (4-5)** | | |
| *Aguilar-Martínez et al.* | 5 | Decreased consumption |
| *Jia et al.* | 4 | Decreased consumption |
| ***Overall: 2 Decreased*** | | |
| **Quality: Low (≤3)** | | |
| *Al Hourani et al.* | 3 | No change in consumption |
| *Konstantinou et al.* | 3 | Unclear |
| *Schwarzova et al.* | 1 | Increased consumption |
| *Androutsos et al.* | 1 | No change in consumption |
| ***Overall: 1 Increased, 2 No Change, 1 Unclear*** | | |

| **Meat, Poultry, and Eggs (n = 8)** | | | |
| --- | --- | --- | --- |
| **Quality: Good (≥6)** | | | |
| ***Author*** | **Quality Rating** | | **Findings** |
| *Cui et al.* | 6 | No change in consumption | |
| ***Overall: 1 No Change*** | | | |
| **Quality: Moderate (4-5)** | | | |
| *Aguilar-Martínez et al.* | 5 | Increased and decreased consumption | |
| *Jia et al.* | 4 | Decreased consumption | |
| ***Overall: 1 Increased, 2 Decreased*** | | | |
| ***Quality: Low (≤3)*** | | | |
| *Al Hourani et al.* | 3 | Increased consumption | |
| *Alfayez et al.* | 3 | Increased consumption | |
| *Konstantinou et al.* | 3 | No change in consumption | |
| *Androutsos et al.* | 1 | No change in consumption | |
| *Schwarzova et al.* | 1 | Increased consumption | |
| ***Overall: 3 Increased, 2 No Change*** | | | |

| **Diet Quality Indices and Overall Dietary Assessments (n=13)** | | |
| --- | --- | --- |
| **Quality: Good (≥6)** | | |
| ***Author*** | **Quality Rating** | **Findings** |
| *Medrano et al.* | 8 | Diet quality increased |
| *Ramos-Alvarez et al.* | 8 | Diet quality increased |
| *Cui et al.* | 6 | Diet quality decreased |
| *Borger et al.* | 6 | No change in diet quality |
| *Husain et al.* | 6 | Diet quality increased |
| *Mastorci et al.* | 6 | Diet quality increased |
| ***Overall: 4 Increased, 1 Decreased, 1 No Change*** | | |
| ***Quality: Moderate (4-5)*** | | |
| *Gedeon et al.* | 5 | Diet quality decreased |
| *Blueher et al.* | 4 | Diet quality decreased |
| *Skolmowska et al.* | 4 | Diet quality increased |
| *Carroll et al.* | 4 | Diet quality decreased |
| *Villodres et al.* | 4 | No change in diet quality |
| ***Overall: 1 Increased, 3 Decreased, 1 No Change*** | | |
| ***Quality: Low (≤3)*** | | |
| *Diaz-Rodriguez et al.* | 2 | Diet quality increased |
| *Nanyakkara et al.* | 1 | Diet quality increased |
| ***Overall: 2 Increased*** | | |

| **Breakfast (n = 14)** | | |
| --- | --- | --- |
| **Quality: Good (≥6)** | | |
| ***Author*** | **Quality Rating** | **Findings** |
| *Kim et al.* | 6 | Decreased consumption |
| *Lee H.A. et al.* | 7 | No change in consumption |
| *Medrano et al.* | 8 | Increased consumption |
| *James et al.* | 5 | Increased consumption |
| ***Overall: 2 Increased, 1 Decreased, 1 No Change*** | | |
| **Quality: Moderate (4-5)** | | |
| *Hanbazaza et al.* | 4 | Decreased consumption |
| *Lee J. et al.* | 5 | No change in consumption |
| *Kyan et al.* | 4 | No change in consumption |
| *Kim et al.* | 4 | Decreased consumption |
| *Moitra et al.* | 5 | Decreased consumption |
| **Overall: 3 Decreased, 2 No Change** | | |
| ***Quality: Low (≤3)*** | | |
| *Konstantinou et al.* | 3 | Decreased consumption |
| *Calabriano et al.* | 2 | Increased consumption |
| *Saltaouras et al.* | 2 | Increased consumption |
| *Angoff et al.* | 1 | Increased consumption |
| *Androutsos et al.* | 1 | Increased consumption |
| **Overall: 4 Increased, 1 Decreased** | | |

| **Snacking (n = 12)** | | |
| --- | --- | --- |
| **Quality: Good (≥6)** | | |
| ***Author*** | **Quality Rating** | **Findings** |
| *Ramos-Álvarez et al.* | 8 | Increased consumption |
| *Szczepanska et al.* | 6 | Increased consumption |
| *Van den Broek et al.* | 6 | Decreased consumption |
| ***Overall: 2 Increased, 1 Decreased*** | | |
| **Quality: Moderate (4-5)** | | |
| *Aguilar-Martínez et al.* | 5 | Increased consumption |
| *Gedeon et al.* | 5 | Increased consumption |
| *Maximova et al.* | 5 | Increased consumption |
| *Carroll et al.* | 4 | Increased consumption |
| ***Overall: 4 Increased*** | | |
| ***Quality: Low (≤3)*** | | |
|  |  |  |
| *Androutsos et al.* | 1 | Increased consumption |
| *Angoff et al.* | 1 | Decreased consumption |
| *Hashem et al.* | 1 | Unclear |
| *Mikulec et al.* | 1 | Increased consumption |
| *Schwarzova et al.* | 1 | Increased consumption |
| ***Overall: 3 Increased, 1 Decreased, 1 Unclear*** | | |

| **Nutrients (n = 5)** | | |
| --- | --- | --- |
| **Quality: Good (≥6)** | | |
| ***Author*** | **Quality Rating** | **Findings** |
| *Perrar et al.* | 9 | Reduced **calories** |
| *Lee H.A. et al.* | 7 | No change in nutrient consumption |
| *Borger et al.* | 6 | Reduced **saturated fat**. Increased **sodium**, **vitamin D**, **calories** |
| ***Overall: Decreased saturated fat. Increased sodium, vitamin D. Mixed findings for calories*** | | |
| ***Quality: Moderate (4-5)*** | | |
| *Kołota et al.* | 5 | Reduced **sugar**, **fat** |
| *Skolmowska et al.* | 4 | Reduced **sugar** |
| ***Overall: Reduced sugar, fat*** | | |
| ***Quality: Low (≤3)*** | | |
| ***Overall: N/A*** | | |
